# Molecular mechanisms and targeted therapy of progranulin in metabolic diseases

**DOI:** 10.3389/fendo.2025.1553794

**Published:** 2025-04-11

**Authors:** Xiaxia Wang, Yonglin Liang, Fan Yang, Yangyang Shi, Ruiwen Shao, Ruge Jing, Tong Yang, Qiao Chu, Dong An, Qi Zhou, Jiayi Song, Haolan Chen, Chun Liu

**Affiliations:** ^1^ School of Basic Medicine, Gansu University of Chinese Medicine, Lanzhou, Gansu, China; ^2^ School of Traditional Chinese and Western Medicine, Gansu University of Chinese Medicine, Lanzhou, Gansu, China; ^3^ TCM Internal Medicine Department, Nanhu Community Health Centre, Pinliang, Gansu, China; ^4^ Library, Gansu University of Chinese Medicine, Lanzhou, Gansu, China

**Keywords:** PGRN, metabolic diseases, inflammation, cartilage repair, bone homeostasis, targeted therapy

## Abstract

Progranulin (PGRN) is a secreted glycoprotein with cytokine-like properties, exerting tripartite mechanisms of inflammation suppression, tissue repair promotion, and metabolic regulation. This multifaceted functionality positions PGRN as a potential “multi-effect therapeutic strategy” for metabolic disorders characterised by cartilage degradation and imbalanced bone remodelling, potentially establishing it as a novel therapeutic target for such conditions. Osteoarthritis, rheumatoid arthritis, intervertebral disc degeneration, osteoporosis, periodontitis, and diabetes-related complications—representing the most prevalent metabolic diseases—currently lack effective treatments due to incomplete understanding of their precise pathogenic mechanisms. Recent studies have revealed that PGRN expression levels are closely associated with the onset and progression of these metabolic disorders. However, the exact regulatory role of PGRN in these diseases remains elusive, partly owing to its tissue-specific actions and context-dependent dual roles (anti-inflammatory vs. pro-inflammatory). In this review, we summarise the structure and functions of PGRN, explore its involvement in neurological disorders, immune-inflammatory diseases, and metabolic conditions, and specifically focus on its molecular mechanisms in metabolic diseases. Furthermore, we consolidate advances in targeting PGRN and the application of its engineered derivative, Atsttrin, in metabolic bone disorders. We also discuss potential unexplored mechanisms through which PGRN may exert influence within this field or other therapeutic domains. Collectively, this work aims to provide a new framework for elucidating PGRN’s role in disease pathogenesis and advancing strategies for the prevention and treatment of metabolic disorders.

## 1 Introduction

In recent years, several new target molecules have emerged with the continuous development of molecular biology. It has been found that changes in the expression of these target molecules significantly impact the progression of diseases, and targeted regulation of these molecules can slow down or even reverse the progression of diseases (1–3). Progranulin (PGRN) is a secreted glycoprotein, also known as granule-epithelial precursor, PC cell-derived growth factor, proepithelial protein (PEPI), GP88 and Acrogranin (4, 5). Owing to its pleiotropic nature, PGRN is widely expressed in various cells, including epithelial cells, neurons, macrophages, immune cells, chondrocytes, neural stem cells, and skeletal muscle (6, 7). Studies have demonstrated that PGRN not only modulates inflammatory processes by regulating TNF-α and its selective binding to receptors TNFR1 and TNFR2 (8, 9), but also enhances extracellular matrix (ECM) synthesis—thereby promoting chondrocyte proliferation and accelerating tissue repair—via activation of pathways such as ERK and PI3K/Akt (10). Furthermore, PGRN maintains the dynamic equilibrium between bone resorption and formation by regulating osteoclast (OC) differentiation and osteoblast (OB) activity (11). Additionally, it participates in glucose and lipid metabolism regulation, ameliorates insulin resistance, and indirectly influences the progression of diabetes-related complications (12, 13). Collectively, these findings suggest that PGRN, through its triple mechanisms of inhibiting inflammation, facilitating tissue repair, and orchestrating metabolic balance, offers a multifunctional therapeutic strategy for metabolic disorders characterised by cartilage destruction and dysregulated bone remodelling. This positions PGRN as a promising novel therapeutic target for the prevention and management of such metabolic diseases.

Osteoarthritis (OA), rheumatoid arthritis (RA), intervertebral disc degeneration (IDD), osteoporosis (OP), periodontitis (EP), and diabetic osteoporosis (DOP) are recognised as major metabolic disorders. Research has shown that the prevalence of these conditions continues to escalate alongside shifts in lifestyle patterns and the progressive ageing of the global population. Current reports indicate: OA affects approximately 300 million individuals worldwide, with annual direct medical costs exceeding USD 150 billion (14, 15). RA has an overall incidence rate of 1–2%, with a 2–3-year disability rate as high as 0.5–1%, and annual direct healthcare expenditures related to RA amount to roughly USD 30 billion (16, 17). IDD exhibits an incidence of 1–5% and a lifetime prevalence of up to 43% (18, 19). OP impacts over 200 million individuals globally (20). EP affects 20–50% of the population (21). Diabetes mellitus (DM) currently afflicts 140 million people, with DOP cases secondary to DM showing a steady annual increase (22). When inadequately managed or progressing to advanced stages, these metabolic disorders are associated with high rates of disability and mortality. They not only diminish patients’ quality of life but also impose substantial burdens on families, socioeconomic systems, and healthcare infrastructure, posing severe threats to public health and societal development (23, 24).Despite advances in research and therapeutic interventions for these conditions, their precise molecular mechanisms remain incompletely elucidated, resulting in a lack of targeted and effective treatment strategies (8, 9, 25).

Recent studies have revealed that in OA patients, PGRN levels in synovial fluid exhibit an inverse correlation with cartilage degradation severity, and exogenous PGRN supplementation mitigates subchondral bone sclerosis and synovitis (26, 27). Furthermore, PGRN competitively binds to TNFR1, blocking TNF-α-induced inflammatory cascades, this mechanism not only reduces joint swelling in collagen-induced arthritis models—demonstrating protective effects against RA—but also alleviates inflammatory responses in EP (28–30). Notably, PGRN-knockout mice develop spontaneous EP phenotypes, accompanied reduction in alveolar bone density (31). In IDD, PGRN expression is markedly downregulated in degenerative disc tissues, adenovirus-mediated PGRN overexpression restores the disc height index normal levels and increases ECM components (32–34). Additionally, PGRN deficiency in murine models leads decline in bone density and elevated OC activity, underscoring its role in suppressing OP (11, 35, 36). Moreover, under high-glucose conditions, PGRN expression is inhibited, impairing OB differentiation, while PGRN overexpression restores osteogenesis and counteracts DOP progression (37).

As demonstrated above, the expression levels of PGRN are closely associated with the progression of metabolic disorders such as OA, RA, IDD, EP, OP, and DOP. However, due to PGRN’s tissue specificity and dual anti-inflammatory/pro-inflammatory effects (38, 39), its precise mechanisms in these metabolic diseases remain incompletely elucidated, which has constrained its clinical translation. By synthesising the molecular mechanisms of PGRN in such disorders and developing strategies for its targeted modulation, this barrier may be overcome, advancing both research and therapeutic applications of PGRN.

This review primarily summarises how PGRN directly or indirectly regulates cartilage degradation, repair, and bone remodelling through diverse pathways, as well as its role in OA, RA, IDD, OP, EP, and diabetes-related complications. Furthermore, targeted regulation of PGRN has been shown to suppress inflammation (directly or indirectly induced), promote cartilage repair, modulate bone remodelling, restore OB-OC equilibrium, and maintain bone homeostasis, thereby offering therapeutic benefits for these metabolic disorders. These insights aim to provide novel research directions and clinical strategies for future studies.

## 2 PGRN overview

### 2.1 Structure and function of PGRN

PGRN is an autocrine growth factor containing 593 amino acid residues with a unique “beaded” structure. This structure consists of 12 highly conserved cysteine-rich motifs (CX5-6CX5CCX8 CCX6CCXDX2HCCPX4CX5-6C) with the sequence P-G-F-B-A-C-D-E (P-P1-G-P2-F-P3-B-P4-A-P5-C-P6-D-P7-E), where A-G is a full repeat sequence and P is a half motif. This structure is essential for maintaining proper protein folding and conformation (40, 41). The secreted full-length PGRN is a highly glycosylated protein with a molecular weight of approximately 90 kDa and anti-inflammatory properties (42, 43). Full-length PGRN can be hydrolysed by various intracellular and extracellular serine (Ser) and threonine (Thr) proteases, such as matrix metalloproteinases and elastases (MMP-9, MMP-12, MMP-14, ADAMTS-7, ELANE, and PRTN3), into homologous subunits with a molecular weight of approximately 6 kDa, known as GRN. These subunits, recognised by 12 cysteine motifs (X2-3CX5-6CX5CCX8CCX6CCX5CCX4CX5-6CX2), perform pro-inflammatory biological functions (44–46), suggesting that the balance between PGRN and GRNs is an important factor influencing the function of PGRN in inflammation. Additionally, PGRN is widely used as a neurotrophic and pleiotropic growth factor (47, 48). Initially found in inflammatory cells and bone marrow, further studies confirmed that PGRN is not only widely expressed in various cells but also functions as a pleiotropic growth factor and is a key participant and regulator in various system functions, such as inflammation, immune response, and tissue regeneration. PGRN plays a crucial role in various diseases (6–9).

### 2.2 Relationship between PGRN and disease

#### 2.2.1 PGRN and neurological disorders

PGRN is a neurotrophic and anti-inflammatory glycoprotein expressed by neurones and microglia in the central nervous system (49). The complexity of PGRN cleavage confers multilayer regulation of various cell types with environmentally responsive processing of PGRN that can change with age and disease (50, 51). However, its functional effects are mainly exerted through extracellular signalling mediated by cell surface receptors, such as Notch, EphA2, and SorCS2, which inhibit related intracellular processes such as TNFR signalling, autophagy, and lysosomal degradation (52). Studies have found that polymorphisms in the GRN gene and the dose of PGRN play a central role in exerting cellular functional effects. Loss of function, mutation of the human GRN gene, and insufficient haploidy of PGRN may potentially affect PGRN levels, leading to neurodegenerative diseases such as frontotemporal degeneration (FTLD), neuronal ceroid lipofuscinosis, and Alzheimer’s disease (4, 53, 54). In addition to neurodegenerative diseases, PGRN deficiency promotes neuroinflammation and apoptosis, aggravating spinal cord injury by exacerbating the release of pro-inflammatory cytokines, such as TNF-α and interleukin (IL)-6, reducing the release of the anti-inflammatory cytokine IL-10, and elevating the expression of iNOS and p-p65 in macrophages/microglia (51). Moreover, PGRN knockdown can exacerbate neuroinflammatory responses, axonal injury, astrocytic proliferation, and neuronal apoptosis caused by astrocytic endoplasmic reticulum (ER) stress (55, 56). Therefore, PGRN is also a potential therapeutic target for diseases such as cerebral ischaemia/reperfusion injury, subarachnoid haemorrhage, acute ischaemic stroke, and nerve damage following spinal cord contusion (39, 57, 58).

In summary, based on the differences in the functional characteristics of PGRN and GRN, this study provides new insights and inspiration for future research on the molecular mechanisms and targeted therapy for the occurrence and development of neurological and other diseases.

#### 2.2.2 PGRN and immune-inflammatory diseases

Regulatory T cells (Tregs) are a subset of CD4+T cells with immunosuppressive effects and play a key role in autoimmune diseases (59). Studies have found that the loss of PGRN leads to a decrease in Treg cell numbers, whereas recombinant PGRN can significantly increase Treg cell numbers (60). Moreover, PGRN dose-dependently promoted CD4+CD25+T cells to CD4+cells under inflammatory conditions *in vitro* through CD25+FOXP3+T cell transformation. In contrast, PGRN deletion leads to the loss of the inhibitory effect of Treg cells on T cell proliferation (61, 62), indicating that PGRN is necessary to maintain Treg function and has anti-inflammatory and immunomodulatory functions.

Additionally, PGRN plays a role in inducing Treg differentiation and inhibiting macrophage chemokine release under inflammatory conditions (63, 64). One study has found that PGRN deficiency upregulated the expression levels of CD4+IFN-γ+Th1 cells, CD4+ IL-17A+Th17 cells, and serum pro-inflammatory cytokines such as IFN-γ, TNF-α, IL-17A, and IL-21, while down-regulating CD4+CD25+Foxp3+ Treg cell levels. In contrast, PGRN treatment decreased the number of Th1 and Th17 cells and increased the number of Treg cells, suggesting that PGRN promotes Treg cell differentiation by inhibiting Th1 and Th17 cell differentiation. These effects are closely related to the occurrence and development of immune-inflammatory diseases, such as RA, inflammatory bowel disease, and psoriasis (60, 65, 66). Conversely, studies have also found that in systemic lupus erythematosus (SLE), loss of PGRN reduces Th1, Th17, IFN-γ, and IL-17A levels, whereas it increases Th2, Treg, Breg, IL-4, and IL-10 levels. This indicates that PGRN enhances Th1 and Th17 differentiation, inhibits Treg differentiation, promotes autoantibody production, and plays a pro-inflammatory role in the development of SLE (67, 68). The role of PGRN in SLE is diametrically opposite to that in RA, suggesting that PGRN’s regulation of T cell function by PGRN may differ depending on the disease state.

Additionally, PGRN deletion led to enhanced natural killer (NK) cell activity, increased NK cell-mediated killing of antiviral T cells, decreased antiviral T cell immunity, and increased viral burden. Conversely, PGRN treatment decreases NK cell expansion, granzyme B transcription, and NK cell-mediated target cell lysis, reduces NK cell cytotoxicity, and improves antiviral T cell immunity (69). These findings suggest that PGRN contributes to the progression of bacterial and viral infections and autoimmune diseases by regulating NK cells.

This review shows that PGRN may be closely related to the occurrence and development of immune-inflammatory diseases, such as RA, SLE, and psoriasis, by interfering with CD4+ T cell differentiation and mediating NK cell activity.

#### 2.2.3 PGRN and metabolic diseases

In recent years, the pleiotropic growth factor PGRN has been widely studied not only in neuroscience but also in oncology because of its powerful anti-inflammatory, neurotrophic, and immunomodulatory functions. PGRN plays a role in tumour pathogenesis and covers a wide range of molecular activities, such as enhancing cell proliferation, migration, invasion, adhesion, and angiogenesis, as well as maintaining cancer stem cells and the tumour microenvironment (70, 71). Current evidence suggests that PGRN mediates several signalling pathways, including PI3K/Akt, ERK1/2, protein kinase C, C-myc, ERK1/2, MAPK, Akt/mTOR, and PGRN/JAK/STAT/PD-1/PD-L1, which regulates the activity of cyclin-dependent kinase 4 and the levels of cyclin B and cyclin D1, upregulates MMP-9 and activates MMP-2, these pathways regulate cleaved caspase-3 (CASP3), nuclear condensation, DNA fragmentation, and poly (ADP-ribose) polymerase (PARP) cleavage; induce angiogenesis, proliferation, invasion, and migration of cancer cells in a sortilin-dependent manner; and inhibit cancer cell apoptosis. PGRN promotes the development of cancers such as colorectal cancer, osteosarcoma, breast cancer, and ovarian cancer (72–75). This suggests that high PGRN expression is associated with poor prognosis and metastasis. Understanding the biological role of PGRN in tumour progression could provide a promising target for developing new cancer therapeutics.

Additionally, increasing evidence shows that PGRN is closely related to metabolic inflammatory bone diseases, as well as to the development of nervous system diseases, immune inflammatory diseases, and tumour diseases. PGRN inhibits chondrocyte degradation and promotes cartilage repair, playing a positive role in cartilage degenerative diseases such as OA, RA, and IDD (5, 10, 11). Conversely, it also promotes OB differentiation, inhibits OC formation and differentiation, regulates inflammation-bone linkage, and maintains bone homeostasis, playing a positive therapeutic role in OP, EP, diabetic OP (DOP), and other bone loss diseases (13, 38).

**Figure 1:**
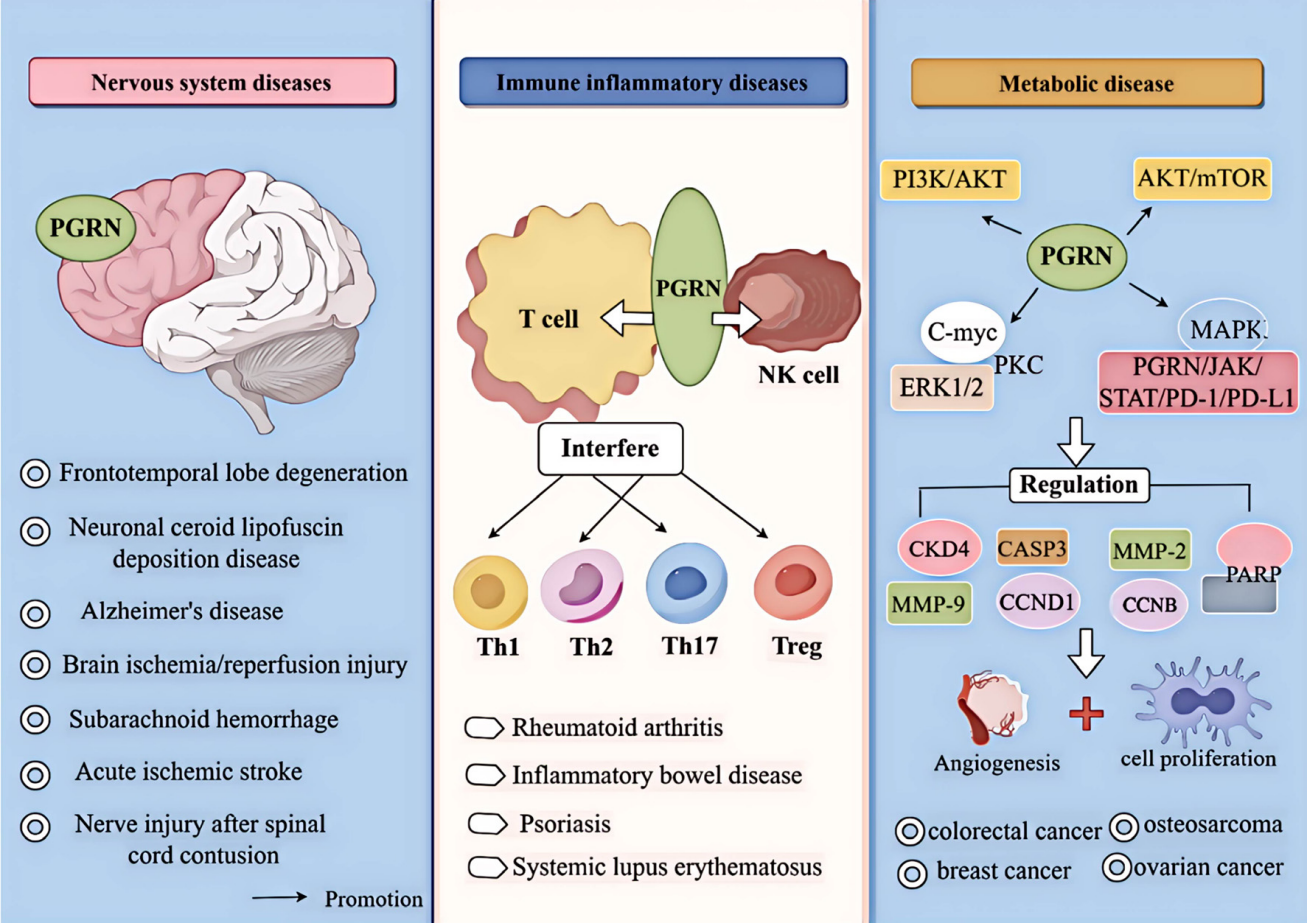
Relationship between PGRN and disease. PGRN is closely related to frontotemporal lobe degeneration, neuronal ceroid lipofuscin deposition disease, Alzheimer’s disease, Brain ischemia/reperfusion injury, subarachnoid haemorrhage, acute ischemic stroke and nerve injury after spinal cord contusion; PGRN is associated with rheumatoid arthritis, inflammatory bowel disease, psoriasis and systemic lupus erythematosus by interfering with T cells (affecting the proliferation and differentiation of Th1, Th2, Th17, Treg, etc.) and NK cells. PGRN mediates PI3K/Akt ERK1/2, PKC, C-myc, MAPK, AKT/mTOR, PGRN/JAK/STAT/PD-1/PD-L1, it can regulate the activity of CKD4 and the levels of CCNB and CCND1, up regulate MMP-9 and activate MMP-2, regulate the cleavage of CASP3 and PARP, and induce the angiogenesis, proliferation, invasion and migration of cancer cells, which is closely related to the development of cancer diseases such as colorectal cancer, osteosarcoma, breast cancer and ovarian cancer.

In summary, PGRN is a double-edged sword with tissue-specific functions. The function of PGRN is tissue-specific, and it is important for the progress of different diseases depending on the systems and environments in which it operates (**Figure 1**). Therefore, by understanding the internal mechanisms driving PGRN and mastering the system in which PGRN is located, targeting PGRN is expected to become a potential strategy for treating specific diseases.

## 3 Molecular mechanisms of PGRN in metabolic diseases

### 3.1 PGRN and OA

OA is an age-related chronic joint degenerative bone disease characterised by progressive destruction of articular cartilage, synovitis, subchondral bone sclerosis, and osteophyte formation. The main clinical manifestations of OA are joint pain, swelling, limited mobility, and joint deformity, which impose a heavy burden on the patient’s life (76, 77). However, the pathogenesis remains unclear. With the development of molecular biology, an increasing number of studies have shown that PGRN, a pluripotent growth factor, is indispensable for cartilage differentiation, repair, and development, as it regulates ER stress (78), autophagy (79), apoptosis (51), and inflammatory (37–39) responses, among others. Therefore, the molecular mechanisms underlying the action of PGRN in OA have been extensively studied.

#### 3.1.1 PGRN regulates OA progression by regulating ER reticulum stress

The ER is a dynamic organelle involved in various cellular functions, including control of lipid metabolism, calcium storage, and protein balance (80). ER stress refers to the characteristic stress response process in which the ER environment is disrupted and protein maturation is impaired during conditions such as hypoxia, nutrient deprivation, oxidative stress, or ageing. This results in the accumulation of misfolded proteins and an unfolded protein response, which interferes with chondrocyte homeostasis (81).

IRE1α is a key molecule in the ER stress signalling pathway, capable of cleaving XBP1 mRNA and regulating the expression of stress response proteins (82). In both *in vivo* and *in vitro* studies, it has been found that overexpression of IRE1α or BMP2 treatment reduced the expression of Col II, SOX9, collagen type X (Col X), MMP-13, Runx2, and IHH and enhanced the expression of parathyroid hormone-related peptide. These findings suggest that IRE1α is a key mediator of chondrocyte hypertrophy. As a new regulator of chondrocyte differentiation, IRE1α inhibits chondrocyte differentiation. However, the study further found that point mutants of IRE1α completely lost the regulatory activities of IRE1α. It has also been found that BMP2 treatment of BMSCs activated IRE1α, and wild-type (WT) IRE1α was able to splice XBP1u and produce XBP1S under ER stress. However, IRE1α point mutants do not splice XBP1u (83), indicating that point mutants that lack enzymatic activity completely lose IRE1α activity. This further suggests that IRE1α-mediated inhibition of cartilage differentiation depends on its enzymatic activity.

**Figure 2:**
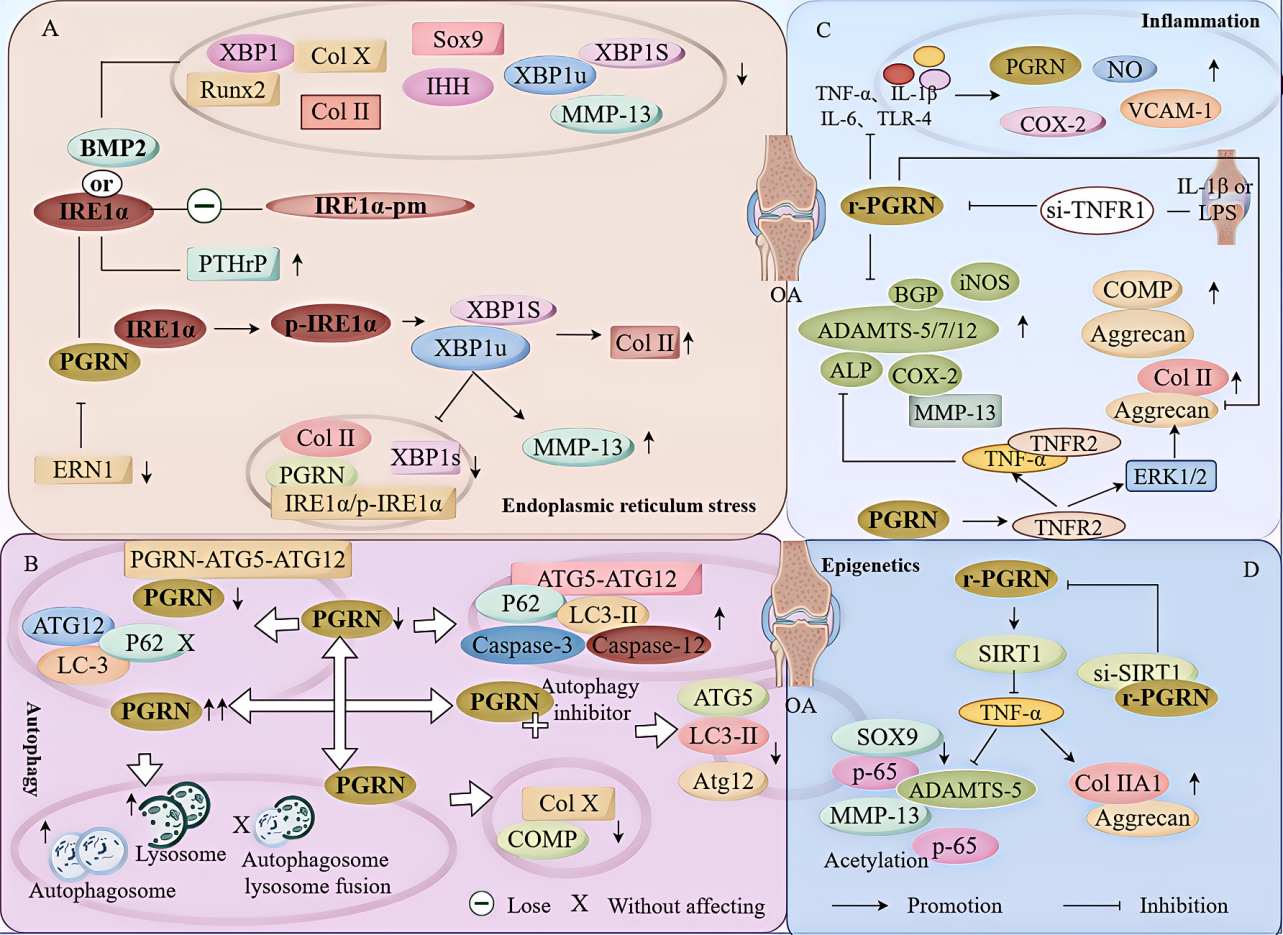
Molecular mechanism of PGRN in osteoarthritis. **(A)** IRE1α overexpression or BMP2 treatment reduced the expression of Col II, Sox9, Col X, MMP-13, Runx2, IHH and increased the expression of PTHrP, while IRE1 α point mutant (IRE1α-PM) completely lost these regulatory activities of IRE1α. PGRN combines with the kinase domain of IRE1α during endoplasmic reticulum stress, assists in IRE1α phosphorylation, promotes XBP1u splicing to generate XBP1s, causes IRE1α to undergo nuclear translocation, and up regulates the expression of Col II. In addition, ERN1 was positively correlated with the level of PGRN. ERN1 deficiency reduced the protein levels of Col II, PGRN, XBP1s and IRE1α/p-IRE1α and increased the number of MMP-13 positive cells by reducing the expression of PGRN and XBP1 splicing, resulting in abnormal collagen structure, which accelerated cartilage degeneration and homeostasis imbalance in OA. **(B)** PGRN deletion significantly increased the expression levels of autophagy markers LC3-II, P62 and ATG5-ATG12 complex, but PGRN deletion did not affect the mRNA levels of LC3, p62 and ATG12. PGRN overexpression increased the level of autophagosome markers and lysosome number, but did not promote autophagosome formation and lysosome biogenesis. PGRN treatment reduced the expression of Col X and COMP, while PGRN and autophagy inhibitor CO treatment down regulated the autophagy marker ATG5, Expression of LC3II and ATG12. PGRN deficiency reduced the interaction between PGRN and PGRN-ATG5-ATG12, increased the expression of P62, Caspase-3 and Caspase-12 in chondrocytes, and decreased the proliferation, increased apoptosis and impaired autophagy activation of chondrocytes. **(C)** The expression of PGRN mRNA and the expression levels of no, COX-2, MMP13 and VCAM-1 in differentiated chondrocytes treated with TNF-α, IL-1β, IL-6 and TLR-4 agonists were significantly increased, while rpgrn significantly inhibited the results induced by inflammatory factors. Under the stimulation of IL-1β or LPS, si-TNFR1 inhibited the anti-inflammatory effect of PGRN. In PGRN deficient mice, the cartilage degeneration related factors ALP, BGP, MMP-13, ADAMTS-5, ADAMTS-7, ADAMTS-12, iNOS and COX-2 were significantly increased, and the degradation rate of COMP and Aggrecan was accelerated, which was reversed by rPGRN. In addition, in a TNFR2 dependent manner, PGRN, on the one hand, increased the levels of anabolic biomarkers Col II and Aggrecan by activating ERK1/2 signalling pathway, and restored the metabolic balance of chondrocytes. On the other hand, PGRN inhibited the expression levels of MMP13, ADAMTS-5, COX-2 and iNOS and the excessive degradation of Col II and Aggrecan by interacting with TNF-α, blocking the progress of OA. **(D)** PGRN enhanced the expression and activity of SIRT1 in a dose-dependent manner, prevented P65 nuclear translocation through p65 deacetylation, inhibited TNF-α induced p65 nuclear accumulation, significantly increased the expression and secretion of Col IIA1 and Aggrecan, and decreased the expression of total p65 and acetylated P65, MMP-13, and ADAMTS-5 in chondrocytes induced by TNF-α. The effect of PGRN was reversed by the CO treatment of rPGRN and si-SIRT1.

Additionally, Liang et al. Have found that ERN1 and IRE1α could enhance chondrocyte viability and inhibit chondrocyte apoptosis, thereby playing a protective role in chondrocytes. PGRN is the main regulator of cartilage metabolism in patients with OA. As an intracellular chaperone protein, PGRN binds to the kinase domain of IRE1α under ER stress, assists IRE1α phosphorylation, promotes XBP1u splicing to generate XBP1s, causes IRE1α to undergo nuclear translocation, upregulates the expression of Col II, maintains collagen homeostasis, and plays a chondroprotective role (84). Additionally, ERN1 was positively correlated with PGRN levels. ERN1 deficiency decreased the protein levels of Col II, PGRN, XBP1s, and IRE1α/p-IRE1α and increased the number of cells positive for MMP-13 by reducing PGRN expression and XBP1 splicing. This results in an abnormal collagen structure, which accelerates cartilage degeneration and homeostasis imbalance in OA (84). These findings indicate that PGRN participates in chondroprotection by mediating ER stress (**Figure 2A**).

#### 3.1.2 PGRN regulates OA progression by regulating autophagy

Autophagy is the primary dynamic degradation mechanism of the circulatory system. The double membrane shed from the ribosome-free attachment area of the rough ER wraps part of the cytoplasm, organelles, proteins, and other components to be degraded in the cell, forming autophagosomes that fuse with lysosomes to form autophagolysosomes, where the wrapped contents are degraded. This process generates new building blocks and energy for cell renewal and homeostasis (85, 86).

It has been found that PGRN deletion significantly increased the expression levels of autophagosome markers LC3-II, p62, and ATG5-ATG12 complexes; however, it did not affect the mRNA levels of LC3, p62, and ATG12 (87). This indicates that the increase in these molecular levels caused by PGRN deletion resulted from post-transcriptional protein accumulation. This suggests that PGRN deletion leads to autophagosome accumulation, damages the fusion of autophagosomes and lysosomes, and inhibits autophagy. It has also been found that PGRN overexpression increased autophagosome marker levels and lysosome numbers; however, it did not promote autophagosome formation or lysosomal biogenesis. Additionally, PGRN overexpression decreases the signal intensity of autophagosome markers or inhibits autophagy flux by reducing autophagosome formation (88). These findings suggest that PGRN overexpression inhibits autophagy flux by inhibiting autophagosome-lysosome fusion and may be a molecular target for regulating autophagy.

In cartilage, chondrocyte autophagy is required to maintain homeostasis, chondrocyte survival, and cartilage integrity. The transition from autophagy to apoptosis plays a key role in the progression of OA (79). Pan et al. Have found that PGRN has an important chondroprotective effect on OA by mediating autophagy activation. In the surgery-induced OA model, intra-articular injection of PGRN significantly protected cartilage from degeneration, whereas injection of autophagy inhibitors significantly inhibited the chondroprotective effect of PGRN. This suggests that the chondroprotective effect of PGRN is closely related to its mediation of chondrocyte autophagy (10). This study has also found that PGRN treatment reduced the expression of Col X and cartilage oligomeric matrix proteins (COMP), whereas co-treatment with PGRN and autophagy inhibitors induced chondrocyte hypertrophy and cartilage degradation markers. Increased expression and downregulated expression of autophagy markers ATG5, LC3II, and ATG12 indicated that PGRN-regulated autophagy contributed to the chondroprotective effect of PGRN in OA (10). Additionally, the study found that the level of the ATG12-ATG5 conjugate is crucial for autophagic activity. PGRN deficiency leads to decreased PGRN and ATG5 expression, reduced PGRN-ATG5 and PGRN-ATG5-ATG12 interaction, and enhanced expression of p62, CASP3, and CASP12 in chondrocytes, whereas the transformation of endogenous LC3 (LC3II/ATG8) is blocked. This results in reduced chondrocyte proliferation, increased apoptosis, and impaired autophagy activation (10).

In summary, PGRN intervenes in OA progression by regulating its molecular phenotype (**Figure 2B**). The occurrence and development of OA are related to ER stress, autophagy, apoptosis, and other phenotypes. This study has also found that pyroptosis, imbalance in mitochondrial homeostasis, and ferroptosis are related to the progression of OA. The protective effect of PGRN in OA is related to its participation in and regulation of pyroptosis, mitochondrial homeostasis imbalance, ferroptosis, and other phenotypes.

#### 3.1.3 PGRN regulates OA progression by regulating inflammation

Inflammation is a normal protective response to pathogen exposure, cellular damage, and stress in both humans and animals. However, dysregulation of the intensity and duration of the inflammatory response can lead to tissue damage (89). For instance, during the occurrence and development of knee OA (KOA), numerous inflammatory factors infiltrate and eventually lead to cartilage destruction through a series of inflammatory reactions (90). As TNF-α is at the peak of the inflammatory cascade, elevated TNF-α levels are associated with inflammation in various diseases. PGRN binds to TNFRs and exerts therapeutic effects in KOA. As found in this study, the expression of TNF-α in the serum of patients with KOA patients increased significantly, and the ratio of PGRN/TNF-α decreased. TNFR1 and TNFR2 act as two receptors for TNF-α, with TNFR1 having a pro-inflammatory effect and TNFR2 having an anti-inflammatory effect. Therefore, the PGRN/TNFR1 pathway promotes inflammation, apoptosis, and catabolism, whereas the PGRN/TNFR2 pathway exhibits anti-inflammatory and anabolic functions (91). This suggests that in KOA, reduction of the PGRN-TNF-α ratio may lead to accelerated catabolism, whereas activation of the PGRN/TNFR2 pathway and/or inhibition of the PGRN/TNFR1 pathway may be key to inhibiting inflammation and rebuilding cartilage balance *in vivo* in patients with KOA.

Additionally, *in vitro* experiments have shown that PGRN mRNA and protein expression increased during chondrocyte differentiation. *In vivo* studies have further found that PGRN expression was significantly increased in human primary chondrocytes, infrapatellar fat pads, and synovium of patients with OA. Treatment of differentiated chondrocytes with pro-inflammatory factors such as TNF-α, IL-1β, IL-6, and TLR-4 agonists significantly increased PGRN mRNA expression, as well as NO, COX-2, MMP13, and VCAM-1 expression levels. In contrast, recombinant PGRN significantly inhibits the effects induced by inflammatory factors (92), indicating that exogenous PGRN counteracts the inflammatory response driven by inflammatory factors. Furthermore, TNFR1 siRNA knockdown inhibited the anti-inflammatory effect of PGRN under IL-1β or lipopolysaccharide stimulation (92), suggesting that PGRN exerts an anti-inflammatory effect by binding to TNFR1. Improving the expression level of PGRN may be a new strategy for the targeted treatment of OA.

Additionally, Zhao et al. Have found that in PGRN-deficient mice, cartilage degeneration-related factors, such as alkaline phosphatase (ALP), BGP, MMP-13, ADAMTS-5, ADAMTS-7, ADAMTS-12, iNOS, and COX-2, were significantly increased. The degradation rate of COMP and Aggrecan was accelerated, which was reversed after recombinant PGRN treatment (93), indicating that PGRN enhanced the anabolism of chondrocytes. The study has further found that PGRN restored chondrocyte metabolic balance by activating the ERK1/2 signalling pathway and raising the levels of anabolic biomarkers Col II and Aggrecan in a TNFR2-dependent manner. In contrast, PGRN inhibited the expression levels of MMP-13, ADAMTS-5, COX-2, and iNOS, as well as the excessive degradation of Col II and Aggrecan, thereby blocking OA progression (93). This indicates that PGRN may mediate anti-inflammatory responses, restore chondrocyte metabolic balance and Extracellular Matrix (ECM) homeostasis, and inhibit cartilage degradation and OA (**Figure 2C**).

#### 3.1.4 PGRN regulates OA progression by regulating epigenetics

Studies have found that PGRN not only regulates ER stress, autophagy, apoptosis, and the inflammatory response but also regulates the deacetylation response, which plays an important role in the progression of OA. For instance, it has been found that treatment of primary rat chondrocytes with recombinant PGRN can significantly increase the expression and secretion of Col IIA1 and Aggrecan, whereas it can attenuate the expression of total P65, acetylated P65, MMP-13, and ADAMTS-5 in TNF-α-induced chondrocytes (94). Further studies have found that PGRN enhanced the expression and activity of SIRT1 in a dose-dependent manner and reduced the acetylation levels of SOX9 and p65, thereby promoting the nuclear translocation of SOX9 and inhibiting TNF-α-induced nuclear accumulation of p65. Co-treatment with transfected SIRT1 reversed this effect of PGRN (94). These findings indicate that PGRN inhibits TNF-α-induced chondrocyte catabolism by activating SIRT1 and subsequently prevents p65 nuclear translocation by deacetylating p65, thereby maintaining chondrocyte homeostasis. PGRN appears to be a promising target for the treatment of OA as well as other cartilage degenerative diseases.

Furthermore, patients with FTLD show increased methylation of the PGRN gene (GRN) promoter and decreased relative expression of GRN. The degree of GRN promoter methylation is negatively correlated with GRN mRNA levels (95). It has also been found that the DNA methylation levels of amplicon A-1 and DNA methyltransferase 3A in the brains of patients with FTLD were significantly increased and both were negatively correlated with the activity and expression of the PGRN promoter (96). In summary, increased methylation of the GRN mRNA promoter in patients with FTLD may lead to decreased GRN expression and play an important role in the epigenetic regulation of PGRN expression. Based on this, we investigated whether the occurrence and development of OA are related to methylation changes in the GRN promoter. Is the occurrence and development of OA a result of epigenetic changes?

In summary, PGRN plays an active role in OA by regulating classical molecular phenotypes or signalling pathways (**Figure 2D**). This not only confirms that the occurrence and development of the disease may be the result of multiple factors but also reflects that a single target molecule can reach the lesion through multiple pathways. However, is the role of PGRN in OA related to the regulation of phenotypes or pathways other than those that have been confirmed or discovered in current studies? Does PGRN play a similar role in other diseases via this pathway? This issue warrants further investigation.

### 3.2 PGRN and RA

RA is a chronic systemic autoimmune inflammatory disease that can occur at any age and is characterised by synovial cell proliferation, inflammatory cell infiltration, and articular cartilage and bone destruction (97). If patients with RA are not treated promptly, persistent inflammation and articular cartilage destruction can lead to synovial hyperplasia, articular cartilage degeneration, and bone erosion as the disease progresses, resulting in permanent disability (98). Although the aetiology and pathogenesis of RA remain largely unknown, it has been shown that PGRN is expressed in various types of cells. The PGRN-TNFR interaction has dual anti-inflammatory and pro-inflammatory properties, and PGRN-TNFR2 binding, which can stimulate PGRN-mediated endochondral osteogenesis, has complex protective effects against inflammatory pathogenic diseases such as RA (65).

#### 3.2.1 PGRN regulates RA progression by regulating inflammation-bone coupling

Clinical studies have revealed significantly elevated PGRN levels in serum and synovial fluid macrophages of RA patients (99). Animal experiments demonstrated that PGRN-deficient mice immunised with type II collagen exhibited more severe arthritis and bone/joint destruction compared to controls (99). Furthermore, research has confirmed PGRN as a downstream molecule of BMP-2 (100), following BMP-2 stimulation in mouse myoblasts (C2C12), marked increases were observed in OB differentiation markers (Col-I, Ocn, Bsp) and ALP activity, TNF-α treatment reversed these BMP-2-induced effects, while PGRN administration blocked TNF-α-mediated suppression of ALP activity and mineralisation (101).

NF-κB, a transcription factor activated in RA, contributes to disease progression by facilitating production of pro-inflammatory cytokines IL-1β and TNF-α (102). Comparative studies revealed that PGRN-treated cells showed significantly enhanced expression of OB-specific markers (ColI, Ocn, Bsp, Runx2, Osx, ATF4), ALP activity, and mineralisation compared to TNF-α/BMP-2 treated cells (101). Notably, PGRN monotherapy substantially reduced NF-κB activity, whereas TNF-α+BMP-2 co-treatment markedly increased it. Crucially, PGRN administration induced concentration-dependent inhibition of NF-κB expression in TNF-α+BMP-2 treated cells (101).

**Figure 3:**
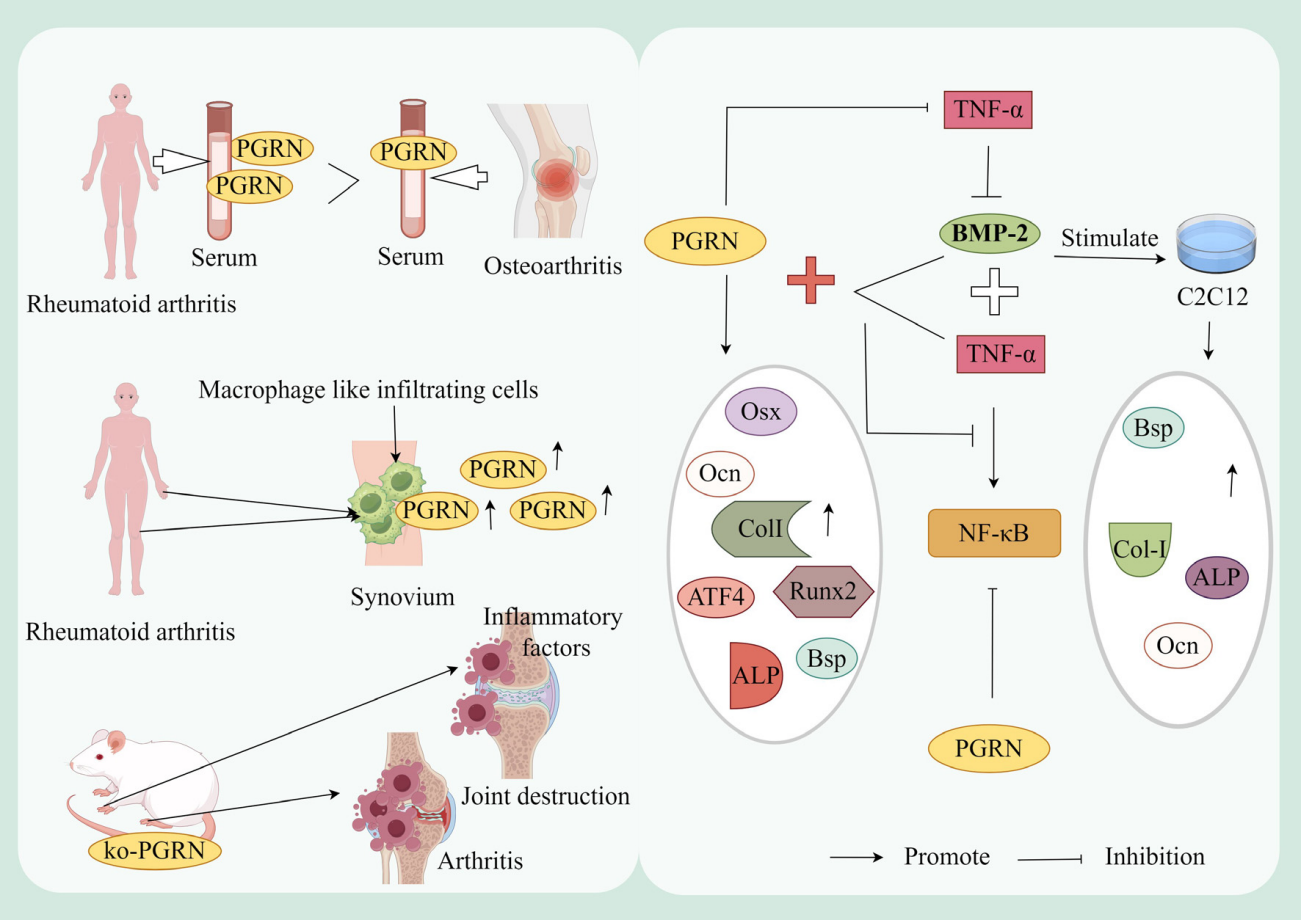
Mechanism of PGRN in rheumatoid arthritis. The levels of PGRN in serum and synovial fluid of RA patients were significantly higher than those of OA patients, and the synovial macrophage like infiltrating cells of RA patients strongly expressed PGRN. In addition, PGRN deficient mice immunised with type II collagen developed more severe inflammatory arthritis and bone and joint damage. The expression of OB differentiation marker genes CoL-I, OCN and BSP and ALP activity were significantly increased after BMP-2 stimulated mouse myoblasts (C2C12). TNF-α treatment reversed this effect of BMP-2. PGRN treatment blocked TNF-α mediated inhibition of ALP activity and mineralisation. Compared with the cells treated with TNF-α and BMP-2 alone, the mRNA expression of OB specific marker genes Col I, OCN, BSP, Runx2, OSX and ATF4, ALP activity and mineralisation of cells treated with PGRN were significantly increased. In addition, the activity of NF-κB was significantly increased after TNF-α+BMP-2 treatment, while the expression of NF-κB was inhibited in a concentration dependent manner after TNF-α+BMP-2+PGRN treatment. In addition, the activity of NF-κB was significantly decreased in cells treated with PGRN alone.

Collectively, these findings indicate that TNF-α-mediated inflammatory responses - both direct and indirect - constitute central mechanisms in RA pathogenesis. PGRN demonstrates therapeutic potential by effectively counteracting TNF-α-driven effects through inflammation-bone coupling mediation and OB differentiation promotion, suggesting its viability as a novel therapeutic target for RA management (**Figure 3**).

### 3.3 PGRN and IDD

IDD is one of the most common degenerative diseases affecting the ageing population. During this process, IVDs undergo extensive morphological and biomechanical changes. Large quantities of inflammatory factors are recruited to drive the inflammatory immune response. Therefore, inflammation is generally considered a major feature of IDD (103, 104). Considering that inflammatory factors play a major role in the IDD process (105), identifying growth factors as markers or potential therapeutic targets is crucial. Recently, it has been reported that PGRN, a chondroprotective growth factor, is closely associated with cartilage-related diseases, such as IDD (32).

#### 3.3.1 PGRN regulates the progression of IDD by regulating inflammation

**Figure 4:**
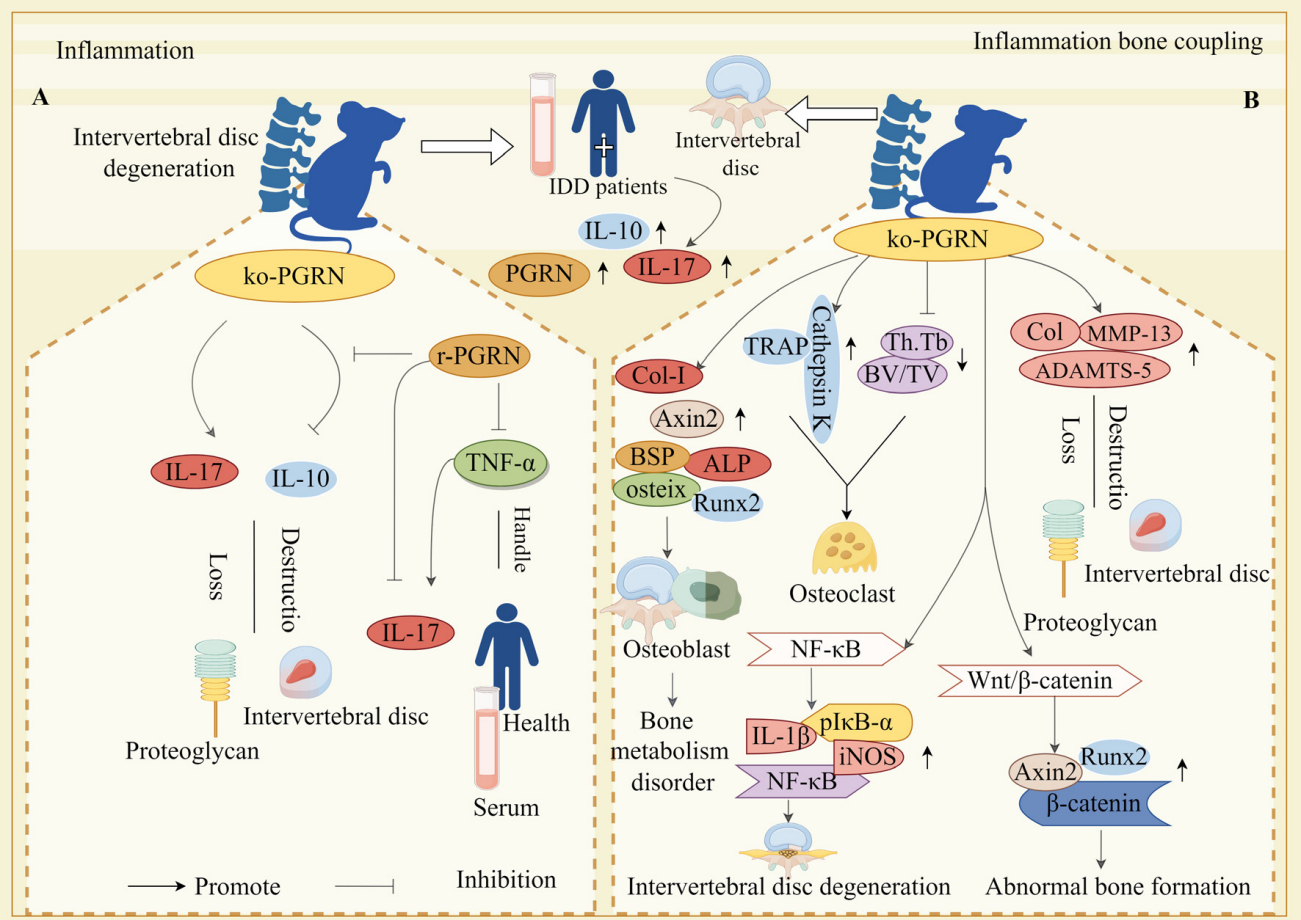
Molecular mechanism of PGRN in intervertebral disc degeneration. **(A)** The expressions of PGRN, IL-10 and IL-17 in peripheral blood and intervertebral disc tissue of IDD patients were significantly increased. In the animal model, the loss of PGRN led to the decrease of IL-10 expression and the increase of IL-17 expression, which accelerated the loss of proteoglycan and the destruction of intervertebral disc. After stimulation with r-pgrn, the transcription level of IL-10 increased significantly. TNF-α significantly increased the transcription and protein levels of IL-17, while r-pgrn significantly inhibited the expression of IL-17 induced by TNF-α. **(B)** The expression levels of OB marker genes ALP, osteix, CoL-I, BSP, AXIN2 and Runx2 were significantly increased in PGRN knockout mice, Bone formation in IVD. The levels of Col, MMP-13 and ADAMTS-5 in cartilage tissue of PGRN-deficient mice were significantly increased, and proteoglycan loss and cartilage degradation in endplate cartilage were serious. The expressions of TRAP and Cathepsin K were significantly increased in the new osteoblasts, BV/TV and Th.Tb decreased significantly, The activity of OC increased. In addition, on the one hand, PGRN deficiency caused the over activation of NF-κB signalling pathway, and the levels of NF-κB, PIκB-α, IL-1β and iNOS were significantly increased, which led to the accelerated degradation of IVD. On the other hand, PGRN deficiency caused the activation of Wnt/β-catenin signalling pathway, and the levels of AXIN2, Runx2 and β-Catenin were significantly increased, which induced abnormal bone formation.

The study revealed that patients with IDD exhibit elevated PGRN, IL-10, and IL-17 expression in both peripheral blood and IVD tissues. PGRN deficiency exacerbated proteoglycan loss and IVD degradation, concomitant with reduced IL-10 and increased IL-17 levels, whereas recombinant PGRN administration restored IL-10 expression (106). Furthermore, experiments demonstrated that treating peripheral blood mononuclear cells from healthy individuals with TNF-α-containing or TNF-α-free media, in the presence or absence of recombinant PGRN, revealed TNF-α-driven upregulation of IL-17, this effect was counteracted by recombinant PGRN treatment (106). These findings collectively suggest that PGRN mitigates inflammatory responses, positioning it as a potential therapeutic target for IDD management (**Figure 4A**).

#### 3.3.2 PGRN regulates the progression of IDD by regulating inflammation-bone coupling

Zhao et al. demonstrated that PGRN is expressed in both human and murine intervertebral disc (IVD) tissues, with expression levels increasing with age. In PGRN-knockout mice, significant upregulation of OB markers (ALP, osteix, Col-I, BSP, Axin2, Runx2) and cartilage-degrading factors (Collagen, MMP-13, ADAMTS-5) was observed, suggesting that PGRN deficiency promotes proteoglycan loss and cartilage degradation alongside enhanced bone formation in IVDs (33). Furthermore, TRAP expression, Cathepsin K levels, and OC activity were markedly increased in newly formed bone and trabeculae of PGRN-deficient mice, accompanied by reduced BV/TV and Th.Tb, concurrently, elevated levels of NF-κB, pIκB-α, IL-1β, iNOS, Axin2, Runx2, and β-catenin in the IVDs of these mice indicated activation of inflammatory signalling (33).

Collectively, these findings reveal a dual role for PGRN: it suppresses inflammatory osteoclastogenesis and preserves cartilage integrity in IVDs by inhibiting the NF-κB pathway; and it attenuates aberrant new bone formation in IVD cartilage by disrupting Wnt/β-catenin signalling-mediated induction of downstream molecules such as Runx2. These mechanisms underscore PGRN’s critical function in preventing dysregulated bone metabolism and cartilage degeneration (**Figure 4B**).

Therefore, OA, RA, and IDD are all cartilage degenerative diseases, and the occurrence and development of OA, RA, and IDD are related to the direct or indirect inflammatory response mediated by TNF-α. This indicates that inflammation remains the core factor in cartilage degeneration and destruction. As a chondroprotective factor, PGRN primarily exerts its effects by inhibiting TNF-α-mediated actions and selectively competing for binding to TNFR (**Table 1**). Consequently, it is a potential therapeutic target for the treatment of degenerative cartilage disease.

**Table 1:** Action mechanism of PGRN in cartilage related metabolic diseases.

Target molecule	Way	Molecular mechanism	Result	Disease	Refs
PGRN	Endoplasmic reticulum stress	The decrease of PGRN expression and XBP1 splicing resulted in the decrease of Col II, PGRN, XBP1s and The expression of IRE1α/p-IRE1α decreased and the number of MMP-13 increased. PGRN combines with IRE1α, assistsp-IRE1α, promotes XBP1u splicing to generate XBP1s, causes IRE1α nuclear translocation, and up regulates Col II.	Maintain collagen homeostasis and play a role in cartilage protection.	OA	(71–73)
	Autophagy	PGRN deficiency reduced the expression of PGRN and ATG5, the interaction between PGRN-ATG5 and PGRN-ATG5-ATG12, and increased the expression of P62, Caspase-3 and Caspase-12, and endogenous LC3 transformation was blocked. Recombinant PGRN reduced the expression of Col X and COMP, while PGRN+autophagy inhibitor induced the expression of markers of chondrocyte hypertrophy and cartilage degradation, and down regulated the expression of ATG5, LC3II and ATG12.	By regulating cartilage autophagy, it promotes the proliferation of chondrocytes and plays a protective role in cartilage.		(76, 77, 79)
	Inflammation	PGRN deletion significantly increased the expressions of ALP, BGP, MMP-13, ADAMTS-5/7/12, iNOS and COX-2, and accelerated the degradation of comp and Aggrecan. Recombinant PGRN increased the levels of Col II and Aggrecan in a TNFR2 dependent manner by activating ERK1/2. PGRN also inhibited the expression of MMP13, ADAMTS-5, COX-2 and iNOS and the excessive degradation of Col II and Aggrecan by interacting with TNF-α.	PGRN restores the metabolic balance of chondrocytes and ECM homeostasis by mediating anti-inflammatory response, and inhibits cartilage degradation.		(80–83)
	Epigenetics	PGRN can increase the expression of Col IIA1 and Aggrecan by activating SIRT1, reduce the acetylation level of SOX9 and p65 induced by TNF-α, promote SOX9 nuclear translocation and inhibit p65 nuclear accumulation	Maintains chondrocyte homeostasis		(84)
	Inflammation-Bone coupling	PGRN significantly increased the mRNA expression of OB specific marker genes Col I, OCN, BSP, Runx2, OSX, ATF4, ALP activity and cell mineralisation, and decreased the expression of TNF-α and NF-κB	Blocking TNF-α mediated activation and promoting OB differentiation	RA	(10, 89, 90)
	Inflammation	The expression of IL-10 was decreased and the expression of IL-17 was increased after PGRN deletion. After recombinant PGRN, the expression of IL-10 was increased and the expression of TNF-α and IL-17 was decreased	The loss of proteoglycan and the destruction of intervertebral disc were inhibited	IDD	(95)
	Inflammation- Bone coupling	PGRN knockout significantly increased the expression levels of ALP, osteix, CoL-I, BSP, AXIN2, Runx2, NF-κB, PIκB-α, IL-1β, iNOS, β-catenin, CoL, MMP-13, ADAMTS-5, TRAP and Cathepsin K, and decreased BV/TV and Th.Tb	Leads to ectopic bone formation, proteoglycan loss, serious cartilage degradation, enhanced OC activity, and disorder of bone metabolism		(96)

### 3.4 PGRN and OP

OP is a systemic metabolic bone disease characterised by a decrease in OB-mediated bone formation and an increase in OC-mediated bone resorption, leading to OB-OC decoupling. This results in imbalanced bone remodelling, decreased bone density and mass, destruction of the bone microstructure, and increased bone fragility (107, 108). With the ageing global population, the incidence of OP is increasing annually, contributing to a large number of bone-related morbidities, mortality, and escalating medical expenses, which place a significant burden on society and families (109, 110). Despite progress in the understanding of OP, the molecular mechanisms underlying its development remain unclear. Previous studies have indicated that factors such as hormones, inflammation, and immune regulation can affect the function of OBs and OCs, thereby influencing the onset and progression of OP (111, 112). In recent years, PGRN has been identified as a factor that can prevent the occurrence of OCs during bone remodelling. By stimulating OB synthesis in new trabecular bone, PGRN demonstrates the ability to promote bone regeneration, which may present a potential therapeutic target for OP (113).

#### 3.4.1 PGRN regulates OP progression by regulating OB differentiation

Osteocytes treated with PGRN responded by rapidly inducing ERK1/2 phosphorylation. However, after incubating osteocytes in serum-free medium for 24 h, the number of viable cells was significantly reduced (114). Conversely, after administering exogenous PGRN, programmed cell death was significantly alleviated at the same concentration; however, this was not reversed, it has also been reported that exogenous PGRN induces MAP phosphorylation in OBs and OB-like cells (114). Several studies have confirmed that risedronate treatment promotes bone formation and has a significant curative effect on OP. Risedronate treatment induces the synthesis and secretion of endogenous PGRN in osteocytes and upregulates PGRN expression (114), further indicating that PGRN is involved in OB differentiation and proliferation, playing an important role in bone growth and bone stress response.

Wang et al. further demonstrated that PGRN-knockout female mice exhibited significantly increased BV/TV, N.Ob/BS, MAR and BFR, while no changes were observed in male counterparts. The study also revealed that OVX WT mice showed significant reduction in L3 trabecular bone BV/TV, whereas PGRN-knockout OVX mice maintained normal levels, indicating PGRN’s critical role in oestrogen deficiency-induced bone loss (115). Moreover, research has established the association between OP pathogenesis and endoplasmic reticulum stress responses within OB cells of OP patients (116). Guo et al. discovered that BMP2 promotes OB differentiation through upregulation of ALP and OCL expression, while overexpression of endoplasmic reticulum-associated factor IRE1α inhibited BMP2-mediated OB differentiation, notably, IRE1α knockdown (siIRE1α) not only significantly enhanced BMP2 expression but also potentiated BMP2-driven OB differentiation (117). Although PGRN has been confirmed to regulate IRE1α expression, its potential to influence OB differentiation and proliferation through IRE1α modulation remains unreported.

**Figure 5:**
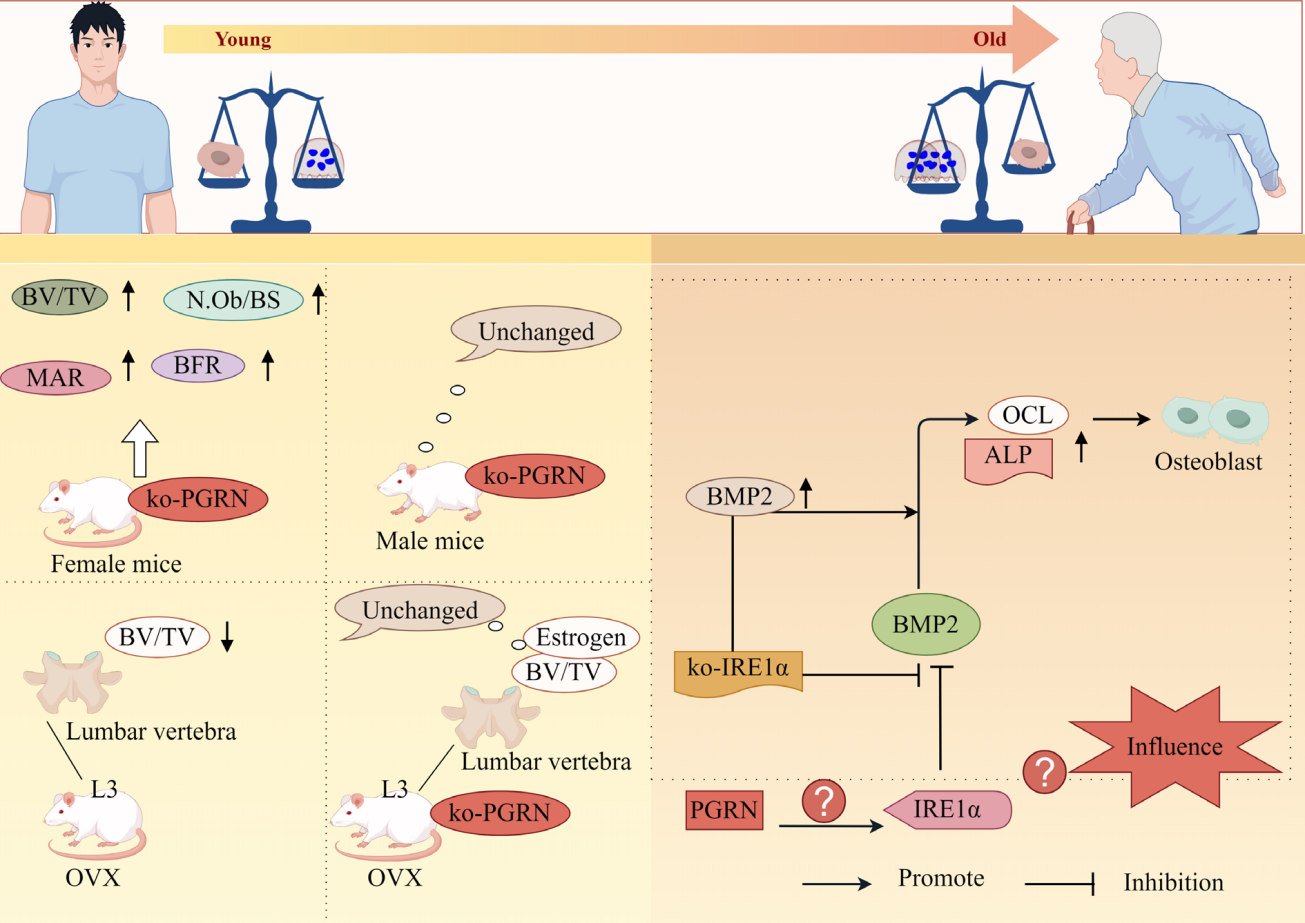
PGRN regulates osteoporosis progression by regulating OB differentiation. Bv/TV, N.Ob/BS, MAR and BFR were significantly increased in PGRN knockout female mice, but not in male mice. The BV/TV of the third lumbar vertebra (L3) cancellous bone in OVX WT mice was significantly decreased, while the BV/TV of the third lumbar vertebra (L3) cancellous bone and oestrogen depletion in OVX ko-PGRN mice were not significantly changed. BMP2 leads to high ALP activity and strong OCL production, and promotes osteogenic differentiation. IRE1α overexpression inhibits BMP2 mediated osteogenic differentiation. IRE1α knockdown can significantly increase the expression of BMP2. ko-IRE1α can increase the expression of ALP and OCL induced by BMP2, and stimulate the differentiation of OB induced by BMP2. Whether PGRN can interfere with OB differentiation and proliferation by regulating the expression of IRE1α has not been reported.

Collectively, these findings suggest that PGRN possesses regulatory functions in OB proliferation and differentiation, potentially offering therapeutic benefits for OP and other bone loss-related disorders (**Figure 5**).

#### 3.4.2 PGRN regulates OP progression by regulating OC differentiation

It has also found that PGRN and E2 can significantly inhibit OC activity, whereas si-PGRN can significantly induce OC activity, indicating that PGRN has the ability to promote osteoclastic activity. Further studies revealed that at low E2 concentrations, PGRN acts as a chaperone protein to assist E2 in binding to E2 receptor α (ERα). However, when the E2 concentration is high, PGRN cannot function as a normal molecular chaperone and interferes with the binding of E2 to ERα, thereby inhibiting osteoclastogenesis (118). Furthermore, studies have confirmed that the PERK/p-eIF2α signalling pathway is one of the important pathways in ER stress and is the primary pathway that promotes osteogenesis and inhibits OC formation (119). ATF4 is a downstream signalling molecule of the PERK/p-eIF2α pathway (120). Both PGRN and E2 significantly upregulate the levels of p-PERK and p-eIF2α. When PGRN and E2 cooperate, the level of p-PERK is higher, and the expression of p-eIF2α and ATF4 is significantly upregulated. Activation of the PERK/p-eIF2α pathway is evident, and the opposite result is observed when ERα and PGRN are knocked out. This suggests that PGRN is required for E2 to regulate bone homeostasis (118).

**Figure 6:**
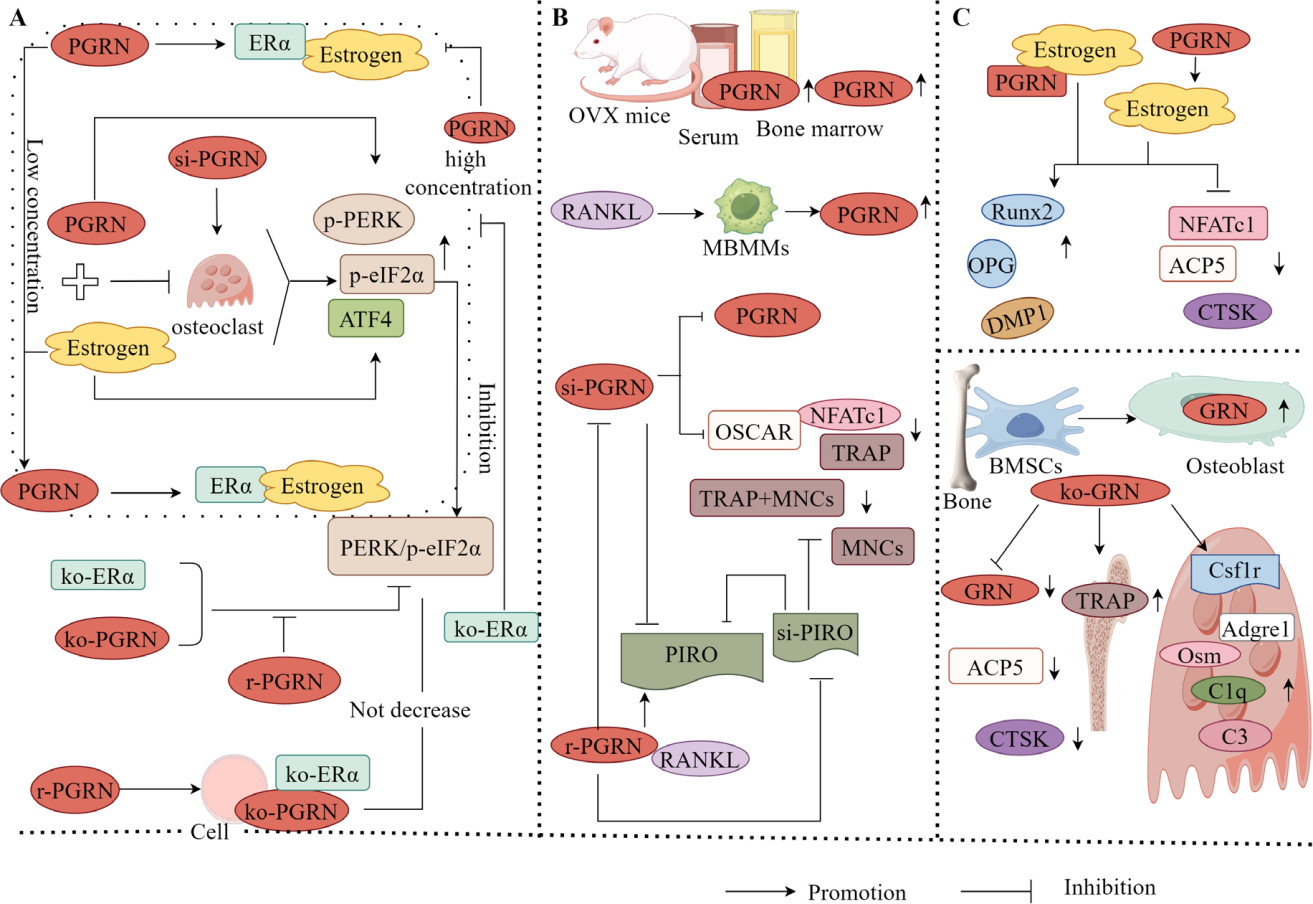
Molecular mechanism of PGRN in osteoporosis. **(A)** PGRN and E2 could significantly inhibit OC activity, while si-PGRN could significantly induce OC activity. At low E2 concentrations, PGRN acts as a chaperone protein to assist the binding of E2 to ERα. However, at high E2 concentrations, PGRN cannot play a normal molecular chaperone role, interfering with the binding of E2 to ERα. The effects of PGRN, E2 and PGRN+E2 on promoting osteogenesis and inhibiting OC were significantly weakened after ko-ERα. PGRN, E2 and PGRN+E2 could significantly up regulate the levels of p-PERK, p-eIF2α and ATF4, and activate the PERK/p-eIF2α signalling pathway. After ko-ERα and ko-PGRN, the results were opposite, while r-PGRN could partially restore the function of PERK/p-eIF2α signalling pathway. In addition, in ko-PGRN cells, after ko-ERα, r-PGRN treatment did not significantly reduce PERK/p-eIF2α signal transduction. **(B)** The levels of PGRN in serum and bone marrow fluid of OVX mice were significantly increased. In RANKL treated mbmms, PGRN mRNA and protein levels were significantly increased in a time-dependent manner. si-PGRN significantly inhibited the gene expression of PGRN, down regulated the expression levels of TRAP, NFATc1 and OSCAR, and reduced the number of TRAP+MNCs. In the presence of RANKL, r-PGRN reversed the above results, and PGRN significantly increased the expression of PIRO, ko-PGRN could significantly reduce the expression of PIRO, while si-PIRO significantly inhibited the formation of MNCs, reduced the number of TRAP+MNCs and PIRO mRNA level, r-PGRN partially restored MNCs inhibited by PIRO transfection. **(C)** PGRN could significantly increase the level of Estrogens, and PGRN and Estrogens could up regulate the expression of osteogenic markers Runx2, OPG and DMP1, and down regulate the expression of OC related markers NFATc1, ACP5 and CTSK. When PGRN+Estrogens co acted, the regulatory effect was more obvious. GRN was expressed in mouse bone tissue and cultured OB from BMSCs. ko-GRN led to a significant reduction in GRN mRNA levels, OC markers CTSK and ACP5 expression in bone and cultured OB from male and female mice with ko-PGRN. In contrast, the expression of TRAP-positive cells, immature OC-associated genes Csf1r, Adgre1 and C1q, and the expression of osteogenesis-related factors C3 and Osm in mouse OC were increased in distal femoral trabeculae.

Additionally, in PGRN knockout cells, using siERα to knock down ERα expression, recombinant PGRN treatment did not significantly reduce PERK/p-eIF2α signal transduction, indicating that ERα is key to PGRN’s regulation of bone homeostasis. PGRN may regulate bone homeostasis by mediating the binding of E2 to ERα through the PERK/p-eIF2α signalling pathway. Moreover, inhibiting the PERK/p-eIF2α signalling pathway blocked the upregulation of osteogenesis-related markers and the downregulation of OC-related markers by PGRN and E2 (118). This indicates that blocking the PERK/p-eIF2α signalling pathway significantly inhibits the function of PGRN and E2, promotes bone formation, and inhibits OC differentiation, confirming that PGRN affects E2 and ERα binding through the PERK/p-eIF2α signalling pathway to regulate bone homeostasis (**Figure 6A**).

PGRN exerts bidirectional regulatory effects on bone homeostasis. PGRN not only promotes osteogenic differentiation and inhibits OC formation alone or as an E2 molecular chaperone but also directly stimulates OC differentiation and accelerates bone resorption *in vivo*. The levels of PGRN in the serum and bone marrow fluid of OVX mice were significantly increased, indicating that the increased expression of endogenous PGRN is associated with bone loss. Further studies have found that in RANKL-treated bone marrow-derived macrophages, PGRN mRNA and protein levels significantly increased in a time-dependent manner. Transfection with PGRN (siPGRN) significantly inhibited the gene expression of PGRN, downregulated the expression levels of TRAP, NFATc1, and OSCAR, and reduced the number of TRAP+multinucleated cells (MNCs). In the presence of RANKL, recombinant PGRN reversed these effects (121), indicating that PGRN is a potent osteoclastic factor in the RANK/RANKL axis. Furthermore, it has been found that PGRN significantly upregulated the expression of PIRO when RANKL was present, and knocking down PGRN significantly attenuated PIRO expression. Transfecting PIRO significantly inhibited the formation of MNCs, reduced the number of TRAP+MNCs, and suppressed PIRO mRNA levels. Recombinant PGRN partially restores PIRO-mediated suppression of MNC formation (121). This suggests that PIRO is the direct target of PGRN in the formation of monocytes (MNCs) and that PGRN and PIRO form a new regulatory axis during OC formation (**Figure 6B**), providing a new therapeutic modality for OP and other bone-related diseases.

#### 3.4.3 PGRN regulates OP progression by regulating OB-OC linkage

For instance, it has been found that PGRN can significantly increase E2 levels. PGRN and E2 can upregulate the expression of osteogenesis-related markers, such as Runx2, OPG, and DMP1, to varying degrees, and downregulate the expression of OC-related markers, including NFATc1, ACP5, and CTSK. When PGRN and E2 act together, their regulatory effects become more pronounced (118). This shows that PGRN has E2-like effects and plays an important role in OB-OC linkage by upregulating osteogenesis-related factors and downregulating OC-related factors. It has also been found that the PGRN gene (GRN) was expressed in mouse bone tissue and BMSC-cultured OBs, *GRN* gene knockout led to a significant reduction in GRN mRNA levels, expression of OC markers CTSK and ACP5, and erosion depth in PGRN knockout male and female mouse bones and cultured OBs, however, the expression of TRAP-positive cells, immature OC-related genes Csf1r, Adgre1, and C1q in the trabecular distal femur of mice, and the increased expression of osteogenesis-related factors C3 and Osm in mouse OCs (115), suggest that GRN directly inhibits bone formation, PGRN produced by OB cells is not responsible for inhibiting bone formation.

It has been shown that PGRN is an important factor in maintaining bone homeostasis and metabolism in female mice. The loss of GRN inhibits the maturation and function of OCs; however, it promotes the production of osteogenic factors in OC cells, an effect that leads to increased bone formation only in females. Therefore, PGRN may be a potential target for the treatment of OP in women.

To sum up, the occurrence and development of OP are mainly owing to the fact that OC-mediated bone resorption is greater than OB-mediated bone formation, leading to OB-OC decoupling and an imbalance in bone homeostasis. Several studies have confirmed that E2 is effective for the treatment of OP. PGRN can be used as a cofactor for E2 to help E2 promote OB differentiation, inhibit OC differentiation, and exert an anti-OP effect. Furthermore, exogenous PGRN has osteoprotective effects, whereas endogenous PGRN has an osteosuppressive effect (**Figure 6C**), indicating that the effects of PGRN are influenced by the internal environment of the body. The influence of the microenvironment on PGRN function should be considered in both basic research and clinical applications.

### 3.5 PGRN and EP

EP is the most common chronic inflammatory disease caused by bacterial infections in the tissues surrounding the teeth. It is associated with destruction of the supporting structures of the periodontal ligament, alveolar bone, and gingival tissue, which may eventually lead to tooth loss (122, 123). Studies have found that the degree and progression of periodontal tissue destruction are related to the host immune-inflammatory response to bacterial attack. Among the complex pro-inflammatory factors, TNF-α is considered the main regulator of EP pathogenesis and is involved in tissue destruction and OC generation in periodontal disease (124). As a regulatory molecule involved in immunity and infection, PGRN plays an important role in various diseases because of its dual function as an anti-inflammatory and pro-inflammatory agent. It has been reported that PGRN can play a protective role in EP-related diseases by antagonising TNF-α or TNFRs (38, 125).

#### 3.5.1 PGRN regulates the progression of EP by regulating inflammation

**Figure 7:**
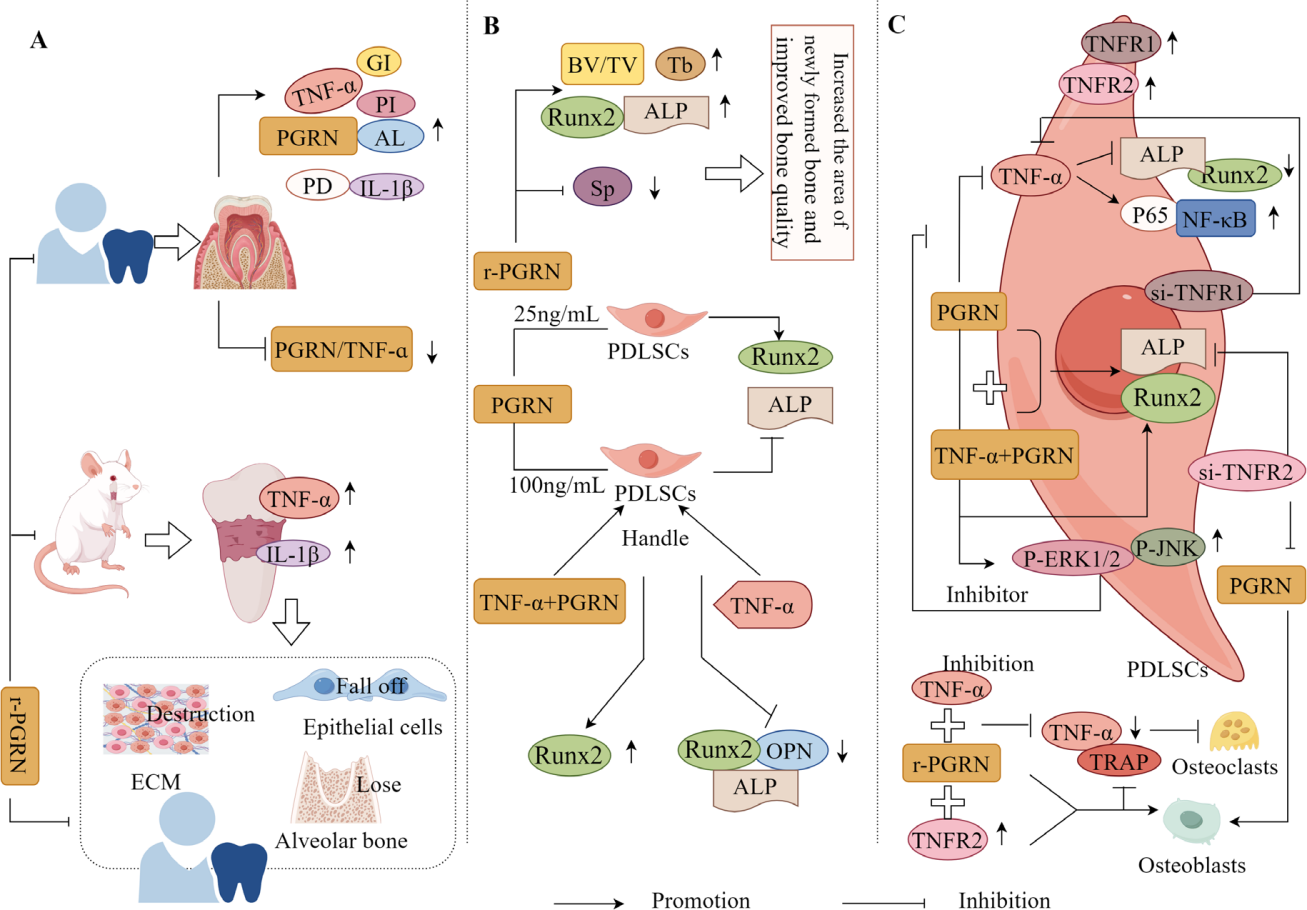
Molecular mechanism of PGRN in periodontitis. **(A)** In CP patients, PI, PD, Al, GI, PGRN, TNF-α and IL-1β in gingiva and gingival crevicular fluid were significantly increased, and PGRN/TNF-α was significantly decreased. The expression of TNF-α and IL-1β in the connective tissue of EP rats were significantly increased, accompanied by ECM destruction, partial epithelial cell loss, and significant loss of alveolar bone. Local treatment with r-PGRN inhibited the inflammatory reaction of periodontal tissue and alleviated the progress of periodontitis. **(B)** r-PGRN can significantly increase the BV/TV and TB values and the expression of ALP and Runx2 in periodontitis rats, reduce the SP value, increase the area of new bone in periodontitis rats, and improve the bone quality. In addition, 25 ng/ml PGRN promoted the proliferation of PDLSCs, significantly increased the expression of ALP and Runx2 mRNA, and promoted the differentiation of OB, while 100 ng/ml PGRN significantly decreased the expression of ALP and Runx2 mRNA. The mRNA and protein expressions of ALP, Runx2 and OPN in PDLSCs treated with TNF-α were significantly decreased, while the mRNA and protein levels of Runx2 in hPDLSCs treated with TNF-α+PGRN were significantly increased. **(C)** TNFR1 and TNFR2 were positive in hPDLSCs. TNF-α stimulation could significantly reduce the expression of ALP and Runx2 in hPDLSCs, and PGRN treatment could significantly reverse the effect of TNF-α. In hPDLSC cells si-TNFR1, the inhibition of osteogenesis mediated by TNF-α disappeared, but TNF-α+PGRN and PGRN alone significantly promoted the mRNA and protein expression of ALP and Runx2. In hPDLSCs cells si-TNFR2, TNF-α-mediated osteogenic inhibition was lost, but both TNF-α+PGRN and PGRN alone significantly promoted mRNA and protein expression of ALP and Runx2. PGRN significantly enhanced the expression of p-ERK1/2, p-JNK, ALP and Runx2 in hPDLSCs si-TNFR1, while ERK1/2 and JNK inhibitors significantly eliminated the expression of ALP and Runx2 enhanced by PGRN. The expression of p65 was significantly increased when hPDLSCs were stimulated with TNF-α. In hPDLSCs transfected with TNFR2, TNF-α activated NF-κB, p65 and inhibited osteogenic differentiation, which was saved by PGRN. In addition, r-PGRN and anti TNF-α treatment can significantly reduce the expression of TNF-α and the number of trap positive cells during periodontal bone defect regeneration, and inhibit OC formation. TNFR2+r-PGRN promotes OB differentiation and inhibits OC differentiation.

Li et al. have found that the levels of periodontal clinical indices, including PI, PD, AL, GI, PGRN, TNF-α, and IL-1β, in the gingival crevicular fluid of patients with Chronic Periodontitis (CP) were significantly increased, and the PGRN/TNF-α ratio was significantly reduced. This change was reversed after one month of non-surgical treatment (with recombinant PGRN), indicating that PGRN/TNF-α in the gingival crevicular fluid of patients with EP was negatively correlated with disease severity and alleviated the progression of EP (126). This suggests that PGRN plays a positive protective role in EP, possibly by antagonising the TNF-α-mediated inflammatory response (**Figure 7A**).

#### 3.5.2 PGRN regulates EP progression by regulating osteoblast differentiation

Recombinant PGRN significantly increased the BV/TV and Tb values, expression of ALP and Runx2, decreased the Sp value, significantly increased the new bone area, and improved bone mass in rats with EP (31). It has also been found that 25ng/mL PGRN promoted periodontal ligament stem cell (PDLSC) proliferation, significantly improved ALP and Runx2 mRNA expression, and promoted OB differentiation, whereas 100ng/mL PGRN treatment significantly reduced ALP and Runx2 mRNA expression. This indicates that an appropriate concentration of PGRN is required to promote PDLSC proliferation and OB differentiation (127). Additionally, the study has also found that the mRNA and protein expression levels of ALP, Runx2, and OPN were significantly reduced in TNF-α-treated PDLSCs, whereas the mRNA and protein levels of Runx2 were significantly increased in TNF-α+PGRN-treated PDLSCs (127). This suggests that PGRN reverses the effect of TNF-α, promoting OB differentiation of PDLSCs and periodontal regeneration under both inflammatory and non-inflammatory conditions (**Figure 7B**).

#### 3.5.3 PGRN regulates EP progression by regulating inflammation-bone coupling

It is well known that PGRN binds to TNFRs with higher affinity than TNF-α, and PGRN can selectively compete for binding to TNFRs (TNFR1/TNFR2); therefore, PGRN exerts different effects by binding to TNFR1 or TNFR2 (128). TNFR1 and TNFR2 were positively expressed in human PDLSCs (hPDLSCs), and TNF-α stimulation significantly reduced the expression of ALP and Runx2 in hPDLSCs, whereas PGRN treatment significantly reversed this result (112). This indicates that PGRN can promote osteogenic differentiation and antagonise TNF-α-mediated inhibition of osteogenic differentiation in hPDLSCs.

Additionally, the study has also found that TNF-α-mediated osteogenic inhibition disappeared when TNFR1 was transfected in hPDLSC cells; however, both TNF-α+PGRN and PGRN alone significantly promoted the mRNA and protein expression of ALP and Runx2 (129). When TNFR2 was transfected in hPDLSCs, the mRNA and protein expression levels of ALP and Runx2 in TNF-α and TNF-α + PGRN groups were significantly reduced, and the bone-promoting effect mediated by PGRN disappeared (129). It was also observed that PGRN significantly enhanced the expression of p-ERK1/2, p-JNK, ALP, and Runx2 in hPDLSCs transfected with TNFR1, whereas ERK1/2 and JNK inhibitors significantly attenuated the expression of ALP and Runx2 enhanced by PGRN, PGRN rescued this process (129). Collectively, these findings suggest that PGRN promotes osteogenic differentiation of hPDLSCs through the dual effects of inhibiting TNF-α/TNFR1/NF-κB and activating the ERK1/2 and TNFR2/JNK pathways (**Figure 7C**). Therefore, PGRN is a potential target for EP treatment and periodontal tissue regeneration.

Chen et al. have also found that recombinant PGRN and anti-TNF-α treatment significantly reduced the expression of TNF-α and the number of TRAP-positive cells during periodontal bone defect regeneration, reduced the periodontal inflammatory response, and inhibited OC production. The therapeutic effect of recombinant PGRN is greater than that of anti-TNF-α treatment, and the activation of TNFR2 plays a protective role in osteogenic differentiation. The interaction between recombinant PGRN and TNFR2 is much stronger than that with TNFR1 (31). These results indicate that PGRN can not only reverse the inhibitory effect of TNF-α on osteogenic differentiation by interfering with the interaction between TNF-α and TNFR1 but may also directly promote osteogenic differentiation by activating the expression of TNFR2, thereby enhancing osteogenic activity under inflammatory conditions and promoting bone healing (**Figure 7C**).

Therefore, the inflammatory response and bone loss caused by the inflammatory response are the main factors in the development of EP. Local or exogenous administration of recombinant PGRN can directly inhibit TNF-α-mediated inflammatory responses or compete with TNFRs to exert anti-inflammatory effects and inhibit bone loss. This, in turn, effectively promotes periodontal bone regeneration and provides a new approach for the treatment of EP (**Table 2**).

**Table 2:** Action mechanism of PGRN in metabolic diseases related to bone homeostasis imbalance.

Target molecule	Way	Molecular mechanism	Result	Disease	Refs
PGRN	OB differentiation	PGRN activated ERK1/2 and MAPK phosphorylation, and promoted ob differentiation and proliferation. Ko-PGRN caused BV/TV, N.Ob/BS, MAR and BFR were significantly increased, but there was no change in male mice	Promote or inhibit bone formation	OP	(104, 105)
	OC differentiation	PGRN upregulated the levels of p-PERK, p-eIF2α and ATF4, activated the PERK/p-eIF2α signalling pathway, and inhibited OC activity. Serum PGRN can promote the expression of TRAP, NFATc1 and OSCAR, increase the number of TRAP+MNCs and promote the differentiation of OC by up regulating the expression of PIRO	Promote or inhibit bone resorption		(33, 109)
	OB-OC coupling	PGRN increased E2 level, and PGRN+E2 up-regulated the expression of OB related markers Runx2, OPG, DMP1, and down regulated the expression of OC related markers NFATc1, ACP5, CTSK. GRN gene up regulates the expression of GRN mRNA and OC markers CTSK, ACP5, TRAP, Csf1r, Adgre1 and C1q.	The absence of GRN inhibits the maturation and function of OC, promotes the production of osteogenic factors in OC lineage cells, and leads to increased bone formation in women		(33, 110)
	Inflammation	PGRN significantly decreased the mRNA and protein levels of TNF-α and IL-1β.	It can inhibit ECM damage, inflammatory reaction of periodontal tissue, reduce inflammatory infiltration, and alleviate the progress of periodontitis	CP	(115)
	OB differentiation	PGRN increased BV/TV, Tb, ALP and Runx2 expression, decreased SP. PGRN at appropriate concentration promoted the proliferation of PDLSCs, increased the expression of ALP and Runx2 mRNA, decreased the expression of TNF-α, and promoted the differentiation of OB.	Increased the area of new bone and improved the quality of bone.		(116, 117)
	Inflammation- Bone coupling	PGRN depended on TNFR2, increased the expression of p-ERK1/2, p-JNK, ALP and Runx2, decreased the expression of TNF-α and the number of TRAP positive cells, and inhibited the formation of OC. PGRN inhibited the expression of TNF-α, NF-κB and p65 depending on TNFR1, and finally inhibited TNF-α/TNFR1/NF-κB pathway and activated ERK, TNFR2/JNK pathway	Reduces periodontal inflammation and promotes OB differentiation		(116, 119)

### 3.6 PGRN and diabetes-related complications

DM is a prevalent non-communicable chronic metabolic disease (130, 131). According to the 2017 Global Statistics of the International Diabetes Federation Atlas, more than 425 million individuals are diagnosed with diabetes. By 2045, this number is expected to increase by 156% (132, 133). As a result, DM has become an increasingly serious public health problem that requires urgent intervention. The two main forms of DM are type 1 (T1DM) and type 2 (T2DM). T1DM is an autoimmune disease characterised by insulin deficiency, whereas T2DM is a chronic metabolic disease caused by multiple aetiologies, including chronic hyperglycaemia (HG), relative insulin deficiency, and insulin resistance (134, 135). T2DM is more prevalent in adults and older individuals, accounting for approximately 90% of all DM cases (136). DM can cause various complications, such as cardiovascular disease (137), nephropathy (138), retinopathy (139), neuropathy (140), and OP (141). Among the several complications of DM, DOP and microangiopathy-diabetic nephropathy (DN) are the most common, but the underlying pathogenic and molecular mechanisms of their occurrence remain unclear.

#### 3.6.1 PGRN and DOP

DOP, a metabolic bone disease, is characterised by decreased bone mineral density, destruction of bone microstructure, and an increased risk of fracture. This severely impairs patient mobility and affects patients physically and socioeconomically (142). Increasing evidence has shown that the incidence and fracture risk of secondary OP in patients are 4–5 times greater than those in the general population (143). Therefore, the prevention and treatment of DOP are of significant economic and social importance. Although the pathogenesis of DOP has not been fully elucidated, inflammation remains a key factor leading to DOP (144, 145). Therefore, inhibition of the inflammatory response is crucial for the treatment of DOP.

##### 3.6.1.1 PGRN regulates DOP progression by regulating inflammation

**Figure 8:**
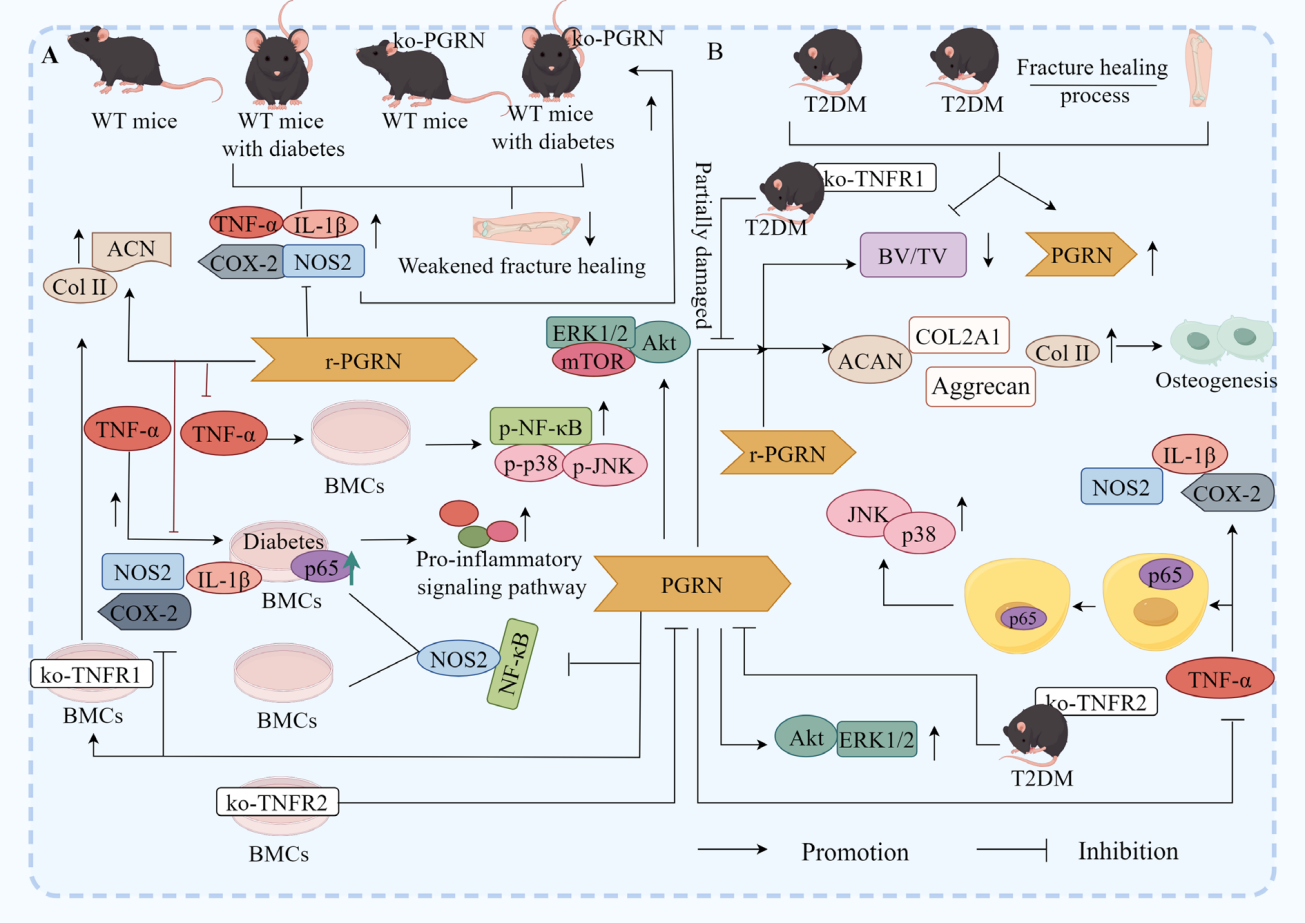
Molecular mechanism of PGRN in DOP. **(A)** Compared with non-diabetic WT mice, the fracture healing of diabetic WT mice, non-diabetic ko-PGRN mice and diabetic ko-PGRN mice was weakened, and TNF-α, IL-1β, NOS2 and COX-2 were significantly increased, especially in diabetic ko-PGRN mice. After r-PGRN treatment, the levels of IL-1β, NOS2, COX-2 and NOS2 were significantly decreased, while Col II and ACN were significantly increased. In normal BMCs, TNF-α induced phosphorylation of P38, JNK and NF-κB, which was inhibited by r-PGRN. In addition, compared with normal BMCs, pro-inflammatory signalling pathway and P65 were over activated in diabetic BMCs, and this activation was further enhanced in the presence of TNF-α, while r-PGRN inhibited TNF-α induced signal transduction in normal and diabetic cells. In normal and diabetic BMCs, PGRN effectively inhibited the activation of NF-κB signal, and the expression of NOS2 increased in the presence of TNF-α, while the up regulation decreased after r-PGRN. In diabetic BMCs, PGRN effectively inhibited the transcription levels of IL-1β, COX-2 and NOS2 induced by TNF-α. In addition, in TNFR1 deficient BMCs, PGRN significantly induced Col II and ACAN transcription levels, while in TNFR2 deficient BMCs, these effects mediated by PGRN were almost eliminated, and Akt, ERK1/2 and mTOR signals were activated by PGRN during PGRN stimulated cartilage formation. **(B)** In the process of fracture healing in T2DM and T2DM, PGRN was significantly increased and BV/TV was significantly decreased. After local administration of r-PGRN, BV/TV ratio was increased, COL2A1, ACAN, Col II and Aggrecan were significantly increased, and bone formation was improved. When TNF-α was present, the expressions of IL-1β, COX-2 and NOS2 were significantly increased, P65 was transferred from cytoplasm to nucleus, and JNK and P38 were activated. PGRN inhibited this reaction mediated by TNF-α, significantly increased the levels of COL2A1 and ACAN in T2DM mice, and activated Akt and ERK1/2 signalling pathways. When ko-TNFR1 was blocked, the anabolic effect of PGRN was abolished, and the effect of PGRN on the activation of Akt and ERK1/2 disappeared.

Notably, the study revealed that compared to non-diabetic wild-type (WT) mice, diabetic WT mice, non-diabetic PGRN-deficient mice, and diabetic PGRN-deficient mice exhibited impaired fracture healing, alongside elevated expression of TNF-α, IL-1β, NOS2, and COX-2, these effects were most pronounced in diabetic PGRN-deficient mice, recombinant PGRN treatment significantly reduced IL-1β, NOS2, and COX-2 expression, increased chondrogenic markers Col II and ACN, accelerated callus formation in diabetic fracture model mice, and enhanced bone formation (**Figure 8A**), indicating PGRN as a critical pro-osteogenic factor (146).

*In vitro* experiments further demonstrated that in normal BMCs, TNF-α induced phosphorylation of p38, JNK, and NF-κB in activated B cells, an effect suppressed by recombinant PGRN, compared to normal BMCs, diabetic BMCs displayed hyperactivation of pro-inflammatory pathways such as p65 and NF-κB, which was further exacerbated by TNF-α, additionally, TNF-α upregulated NOS2 expression in both normal and diabetic BMCs, a process attenuated by recombinant PGRN (146). Notably, in diabetic BMCs, PGRN effectively inhibited TNF-α-induced transcriptional upregulation of pro-inflammatory cytokines IL-1β, COX-2, and NOS2, irrespective of PGRN presence or absence during stimulation (146). The study also found that in TNFR1-deficient BMCs, PGRN markedly induced transcriptional levels of Col-II and ACAN, whereas these effects were nearly abolished in TNFR2-deficient BMCs, furthermore, during PGRN-stimulated chondrogenesis, Akt, ERK1/2, and mTOR were activated (146).

These findings suggest that PGRN suppresses inflammatory responses and induces osteogenesis via the TNFR2-Akt/ERK1/2/mTOR pathway, thereby promoting fracture healing in diabetic models (**Figure 8A**).

##### 3.6.1.2 PGRN regulates DOP progression by regulating inflammation-bone coupling

Ding et al. have found that under T2DM conditions and during T2DM fracture healing, PGRN transcription and protein levels were significantly increased, whereas the bone volume fraction (BV/TV) was significantly reduced (147). After local administration of recombinant PGRN, the BV/TV ratio was effectively increased, and the transcription levels of COL2A1 and ACAN, along with their encoded chondrogenic biomarkers, Col II and Aggrecan, were significantly increased. Furthermore, callus formation was reduced, whereas the cartilage area, proportion, and new bone proportion increased, leading to improved bone formation. The addition of PGRN inhibits TNF-α-mediated responses (147). PGRN also significantly increased the levels of the cartilage anabolic biomarkers COL2A1 and ACAN in T2DM mice and activated the Akt and Erk1/2 signalling pathways. When TNFR1 was blocked, the expression of COL2A1 and ACAN was partially impaired, but the effect of PGRN on Akt and Erk1/2 activation remained unchanged, when TNFR2 was blocked, the anabolic effect of PGRN was cancelled, and the effect of PGRN on Akt and Erk1/2 activation disappeared (147), indicating that PGRN inhibited the inflammatory response, increased bone formation, and promoted fracture healing in T2DM through the TNFR2, Akt, and Erk1/2 pathways (**Figure 8B**).

Taken together, these findings demonstrate that PGRN inhibits TNF-α-mediated effector mechanisms by dependence on TNFR2, activates the TNFR2-Akt/ERK1/2 signalling pathway, and plays a role in inhibiting inflammation and promoting bone formation.

#### 3.6.2 PGRN and DN

DN is a major microvascular complication of DM and the most common cause of end-stage renal disease (148, 149). Over the last decade, several studies have shown that inflammation is a key process in the occurrence and development of diabetes and its microangiopathies. In this context, PGRN have attracted considerable attention. As an adipocytokine, PGRN plays an important role in the regulation of inflammatory events (150, 151).

##### 3.6.2.1 PGRN regulates DN progression by regulating inflammation

**Figure 9:**
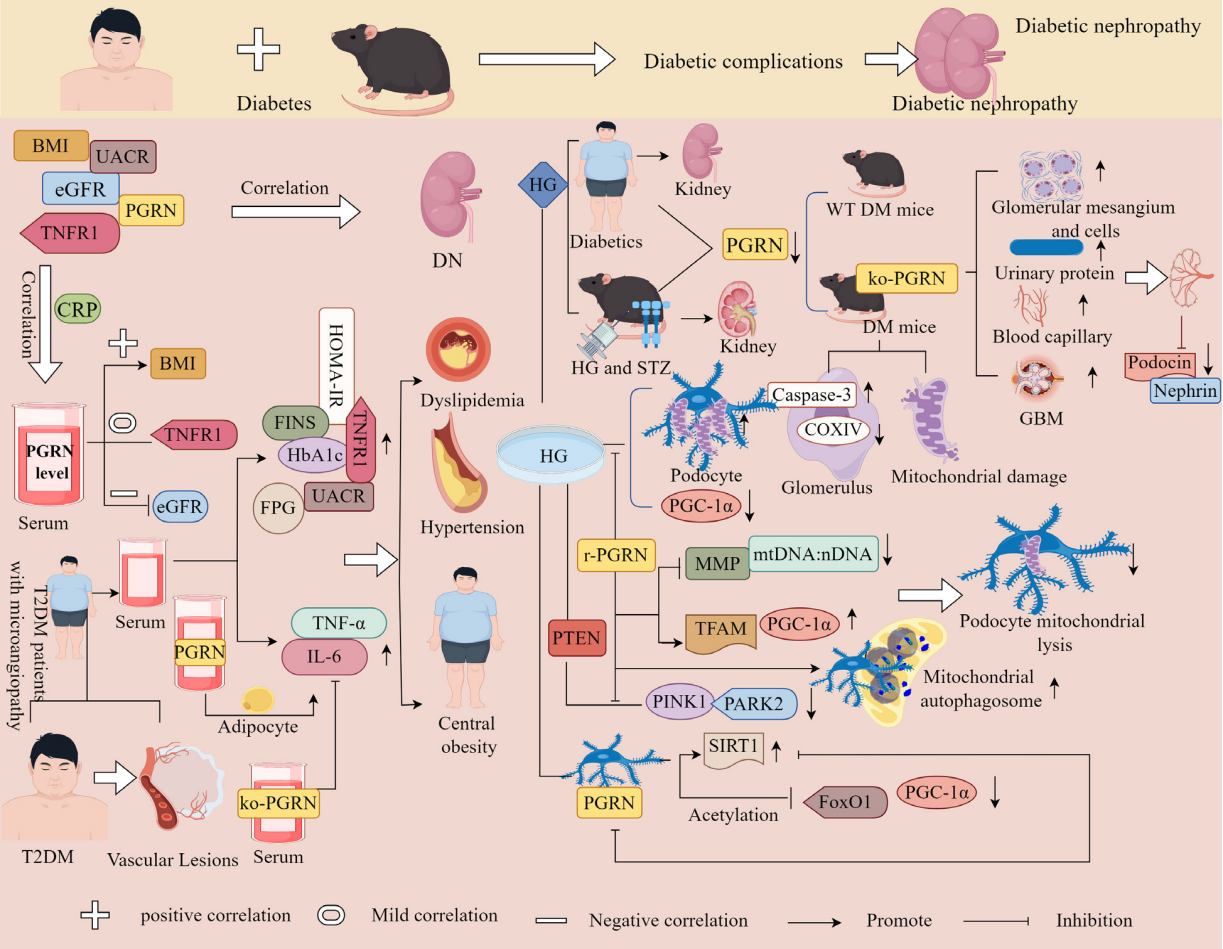
Molecular mechanisms of PGRN in diabetic nephropathy. BMI, EGFR, UACR, TNFR1 and PGRN were correlated with the occurrence of DN, and serum PGRN level was correlated with BMI, CRP, EGFR and UACR. Serum PGRN level was positively correlated with BMI, negatively correlated with EGFR, and slightly correlated with TNFR1. The level of PGRN in T2DM patients with microangiopathy gradually increased, and the values of HbA1c, FPG, FINS, HOMA-IR, TNF-α and IL-6 significantly increased, and dyslipidaemia, hypertension and central obesity occurred. In addition, serum PGRN can stimulate adipocytes to release more IL-6, while ko-PGRN can completely block IL-6 secreted by TNF-α. The level of PGRN in renal biopsy tissue of DN patients decreased. Decrease of PGRN level in kidney of HG and STZ induced diabetic mice, compared with WT type diabetic mice, PGRN deficient diabetic mice had glomerular mesangial dilation, increased cells, increased urinary protein excretion, increased capillaries and GBM foot process loss, and further decreased glomerular capillary loop slit membrane proteins Podocin and Nephrin. In diabetic mice with PGRN deficiency, caspase-3 was increased, coxiv was decreased, and mitochondrial damage was aggravated. Under HG condition, the percentage of mitochondrial fragmentation in podocytes was higher, PGC-1α was significantly reduced, r-PGRN significantly inhibited l HG induced mitochondrial division, reduced the decline rate of MMP, mtDNA: nDNA ratio, and enhanced the protein expression levels of PGC-1α and TFAM. PINK1 and PARK2 were significantly decreased under HG conditions, which was rescued by r-PGRN, and mitochondria and autophagosomes co-localised in r-PGRN-treated podocytes. In HG-treated podocytes, PGRN specifically induced the expression of SIRT1 and reduced the acetylation levels of PGC-1α and FoxO1, whereas the inhibition of SIRT1 expression counteracted the protective effect of PGRN on podocytes.

Studies have confirmed that BMI, glomerular filtration rate (eGFR), UACR, TNFR1, and PGRN levels are significantly related to the risk of renal complications, whereas serum PGRN levels are related to BMI, CRP, eGFR, and UACR. Serum PGRN levels are positively correlated with BMI, negatively correlated with eGFR, and slightly correlated with TNFR1 levels (152), indicating that PGRN is closely related to kidney disease and its complications, with the inflammatory response potentially serving as a link or bridge. The study has found that the level of PGRN in the serum of patients with T2DM complicated by microangiopathy gradually increased, while the values of HbA1c, FPG, FINS, HOMA-IR, and inflammatory markers, such as TNF-α and IL-6, were significantly increased. Additionally, dyslipidaemia, hypertension, overweight, and central obesity were observed (153). This study has further found that PGRN levels were significantly and positively correlated with inflammatory markers, systolic and diastolic blood pressure, BMI, TG, and UAER, suggesting that PGRN is associated with obesity, lipid metabolism disorders, and hypertension. Additionally, it has also been found that serum PGRN can stimulate adipocytes to release more IL-6, whereas ablation of the PGRN gene completely blocked the increased secretion of IL-6 induced by TNF-α (153). This suggests that PGRN levels are closely related to the pro-inflammatory state that is common in diabetic microangiopathy (**Figure 9**). Therefore, PGRN may serve as a biomarker for chronic inflammatory responses in T2DM microangiopathy.

##### 3.6.2.2 PGRN regulates DN progression by regulating autophagy

Furthermore, it has been found that podocytes lose specific differentiation markers, undergo foot process regression and detachment, and lose the ability to maintain the glomerular filtration barrier in diabetic settings, leading to proteinuria. This suggests that podocyte injury may be a major determinant of the prognostic development of DN (153). Therefore, preventing podocyte injury or promoting podocyte repair is a potential strategy for DN treatment.

Zhou et al. discovered that PGRN levels were reduced in the kidneys of DN patients and in HG- and streptozotocin (STZ)-induced diabetic mice, compared to WT diabetic mice, PGRN-deficient diabetic mice exhibited elevated urinary protein levels and Caspase-3 expression, alongside pathological features including mesangial expansion, increased cellularity and capillarisation, loss of podocyte foot processes in the GBM, reduced COXIV expression, and decreased slit diaphragm proteins podocin and nephrin in capillary loops (154).

*In vivo* and *in vitro* experiments further revealed that under HG conditions, podocytes displayed a higher percentage of mitochondrial fragmentation and diminished PTEN-induced expression of PINK1, PARK2, and PGC-1α. Recombinant PGRN suppressed HG-induced mitochondrial fission, attenuated the decline in MMP and the mitochondrial-to-nuclear DNA ratio (mtDNA:nDNA), and promoted mitochondrial-autophagosome colocalisation in podocytes (154). Additionally, PGRN increased the number of mitophagic vacuoles and upregulated PGC-1α and TFAM expression. Notably, PGRN specifically induced SIRT1 expression, reducing acetylation levels of PGC-1α and FoxO1, whereas SIRT1 inhibition abolished PGRN’s protective effects on podocytes (154). These findings collectively demonstrate that PGRN maintains mitochondrial homeostasis and restores podocyte structure and function through the PGRN-SIRT1-PGC-1α/FoxO1-mediated mitochondrial biogenesis and mitophagy, suggesting a novel therapeutic strategy for diabetes mellitus (**Figure 9**).

Zhou et al. Have also demonstrated that in STZ-induced diabetic mice kidneys, PGRN expression was decreased while the kidney weight-to-body weight ratio significantly increased. Pathological changes included mesangial expansion, capillary atrophy, thickened GBM, widened and effaced podocyte foot processes, along with reduced LC3II/I expression and aggravated podocyte autophagy impairment. These alterations were reversed by recombinant PGRN administration (155). *In vitro* experiments further revealed that under HG conditions, PGRN expression decreased in a concentration- and time-dependent manner in human podocytes (HPCs), mouse podocytes (MPCs), and human renal proximal tubular HK2 cells, accompanied by reduced cleavage of Caspase-3 and PARP1 effects counteracted by recombinant PGRN treatment. These findings suggest PGRN promotes podocyte autophagy in diabetic mice (155).

**Figure 10:**
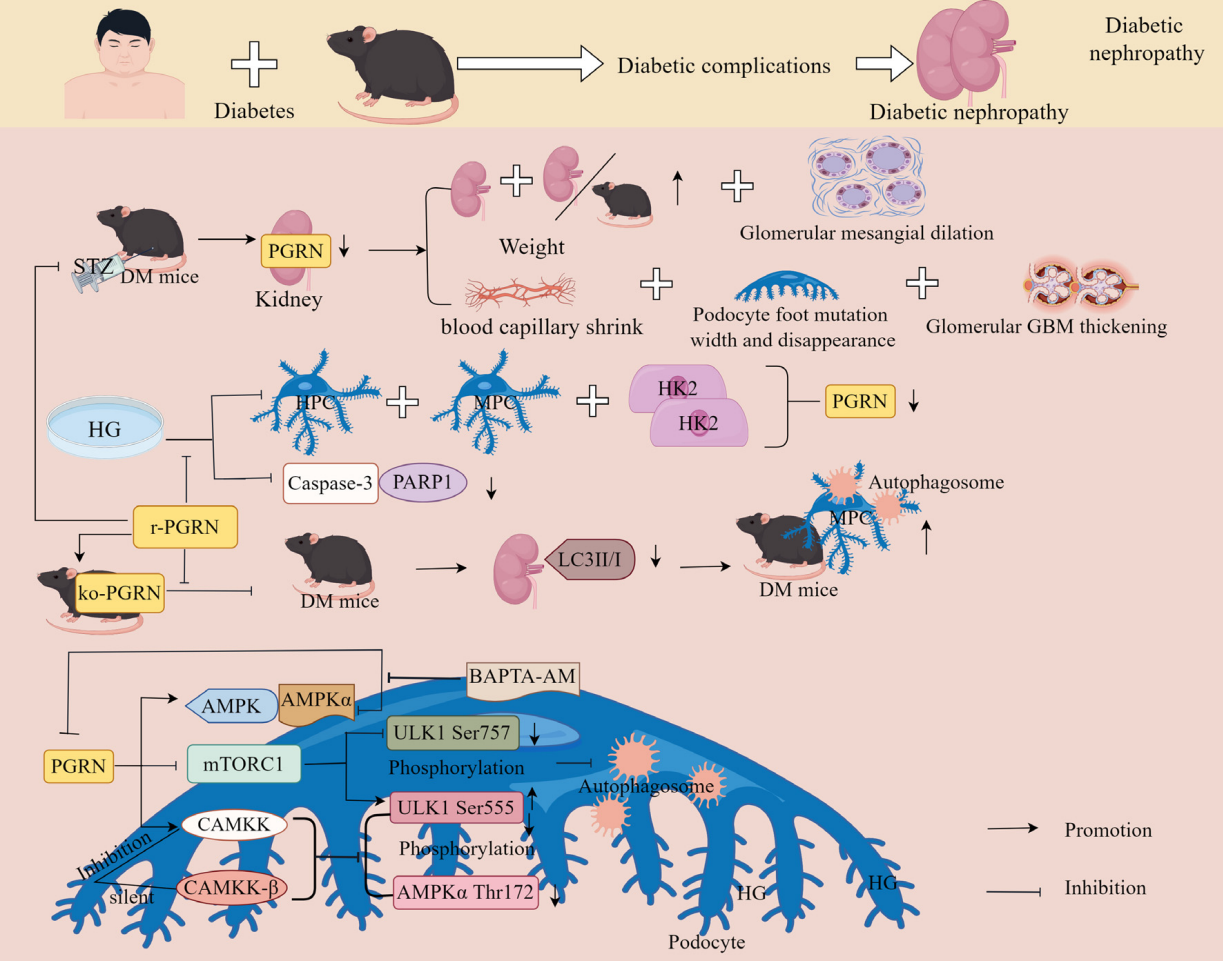
Molecular mechanisms of PGRN in diabetic nephropathy. PGRN expression was significantly reduced in the kidneys of STZ-induced diabetic mice, and PGRN deficiency resulted in significantly higher kidney weights, kidney weight to body weight ratios, dilation of glomerular tunica and capillary atrophy, thickening of the glomerular GBM, and widening and loss of podocyte pedicle synapses, which were reversed by r-PGRN. Under HG conditions, PGRN expression was reduced in human podocytes (HPC), mouse podocytes (MPC), and human renal proximal tubule HK2 cells, and cleavage of caspase-3 and PARP1 was reduced, with the opposite result for r-PGRN treatment. *In vivo*, ko-PGRN decreased the ratio of LC3II/I and aggravated autophagy damage in the kidneys of diabetic mice foot cells; *in vitro*, r-PGRN treatment was the opposite. PGRN activates AMPK signalling and inhibits mTORC1 activity in HG-treated podocytes, resulting in enhanced ULK1 Ser555 phosphorylation sites and reduced ULK1 Ser757 phosphorylation sites in podocytes, whereas inhibition of the AMPK signalling pathway impairs PGRN-rescued autophagy and podocyte survival under hyperglycaemic conditions. CAMKK is activated by PGRN in podocytes under HG condition, silencing of the CAMKK-β gene or specific inhibition of CAMKK activity reduces PGRN-mediated phosphorylation of the AMPKα Thr172 phosphorylation site and ULK1 Ser555 phosphorylation site, and BAPTA-AM chelating of the cytoplasmic calcium-free antagonises the PGRN-activated AMPKα signalling pathway.

The study additionally uncovered a dual mechanism: PGRN activates AMPK signalling while inhibiting mTORC1 activity in HG-treated podocytes, enhancing ULK1 Ser555 phosphorylation and reducing ULK1 Ser757 phosphorylation to induce autophagy, AMPK pathway inhibition compromised PGRN-mediated autophagy restoration and podocyte survival under HG conditions; PGRN activates CAMKK in HG-stressed podocytes, whereas CAMKK-β silencing or specific CAMKK inhibition attenuated PGRN-induced phosphorylation of AMPKα Thr172 and ULK1 Ser555. Cytosolic calcium chelation with BAPTA-AM antagonised PGRN-activated AMPKα signalling (155). Collectively, these results indicate that PGRN exerts protective effects against podocyte injury through autophagy restoration and CAMKK/AMPK pathway activation, potentially representing a novel therapeutic approach for diabetes mellitus (**Figure 10**).

PGRN has shown a positive therapeutic effect on diabetic complications such as DOP and DN by inhibiting inflammatory responses and other effects triggered by inflammation (e.g. bone homeostasis imbalance and autophagy disorders) (**Table 3**). However, in addition to DOP and DN, diabetic complications include diabetic cardiovascular disease, diabetic cerebrovascular disease, diabetic retinopathy, diabetic foot disease, and diabetic neuropathy. Are their occurrence and development related to changes in PGRN expression? Are they also related to PGRN-mediated inflammatory responses, autophagy, or mitochondrial autophagy? This issue warrants further investigation.

**Table 3:** Mechanism of PGRN in diabetic complications.

Target molecule	Way	Molecular mechanism	Result	Disease	Refs
PGRN	Inflammation	PGRN+TNFR2: Activated Akt, ERK1/2 and mTOR, reduced the transcription levels of TNF-α, IL-1β, NOS2 and COX-2 and NOS2 protein levels, up-regulated the expression of col II and ACN, and inhibited the phosphorylation of p38, JNK and NF-κB induced by TNF-α.	By inhibiting the inflammatory reaction, the bone formation increased and the residual bone space decreased, which promoted the healing of DOP.	DOP	(134)
	Inflammation-Bone coupling	PGRN+TNFR2: Activated Akt and ERK1/2,reduced the expression of TNF-α, IL-1β, COX-2 and NOS2, inhibited p65 translocati-on and activation of JNK and p38, increa-sed BV/TV ratio, transcriptional levels of COL2A1 and acan, and the expression of COL-II and Aggrecan.	The area and proportion of cartilage and the proportion of new bone were increased, and bone formation was improved.		(135)
	Inflammation	Serum PGRN levels were positively correlated with TNF-α, IL-6, SBP, DBP, BMI, TG, UAER and BMI, and negatively correlated with EGFR	Serum PGRN is closely related to the occurrence of diabetic microangiopathy by promoting Inflammation response.	DN	(140, 141)
	autophagy	PGRN defect: Caspase-3, complex IV and COXIV decreased. The recombinant PGRN activated SIRT1, up-regulated PINK1 and PARK2, inhibited HG induced mitochondrial division, reduced the decline rate of MMP and mtDNA:nDNA, reduced the acetylation of PGC-1α and FoxO1, and enhanced the protein expression of PGC-1α and TFAM. PGRN increased the cleavage of Caspase-3 and PARP1 and the proportion of LC3II/I by mediating CaMKK to activate AMPK, inhibited mTORC1 activity, increased p-ULK1 ser555 site and decreased p-ULK1ser757 site in podocytes.	Promote podocyte mitochondrial homeostasis, induce podocyte autophagy, reduce cell apoptosis, improve podocyte injury in DN mice, and protect renal function.		(142, 143)

## 4 Therapeutic role of targeted regulation of PGRN in metabolic diseases

### 4.1 Mechanism of non-coding RNA targeted regulation of PGRN in OA

MicroRNAs (miRNAs) are small double-stranded non-coding RNA molecules, typically 20–25 nucleotides in length, that play a central role in the post-transcriptional regulation of protein-coding genes by inhibiting messenger RNA translation or promoting mRNA degradation (156, 157). It has been reported that miRNAs are powerful regulators of osteocyte growth, differentiation, development, and apoptosis and play an important role in the occurrence and development of metabolic bone diseases (158, 159). For instance, Chen et al. have found through *in vivo* and *in vitro* studies that both miR-29b-3p and PGRN expression in OA cartilage tissue and cells were upregulated. miR-29b-3p mimetics significantly inhibited the expression and release of PGRN, increased the expression of Bax, CASP3, MMP-1, MMP-13, and COL X, and inhibited the expression of Bcl-2. These findings indicate that miR-29b-3p promotes OA progression by negatively regulating PGRN (160). It has also been found that matrine can reduce the production of the pro-inflammatory cytokines IL-6, IL-1β, and TNF-α in IL-1β-treated chondrocytes, inhibit miR-29b-3p expression, and increase PGRN expression, Matrines also improve chondrocyte viability, inhibit apoptosis, and alleviate joint tissue destruction, suggesting that matrine exerts its anti-OA effects by regulating the miR-29b-3p/PGRN axis (161). In summary, miR-29b-3p was positively correlated with the occurrence and development of OA, and its promotion of OA was achieved by inhibiting PGRN expression.

Long non-coding RNA (lncRNAs) are transcripts over 200 nucleotides in length with no or very limited protein translation capacity, it is one of the largest and most significantly diverse families of RNA that has emerged in recent years (162, 163). lncRNAs not only play an important role in the regulation of osteogenesis-osteoclastogenesis coupling but can also act as competitive endogenous RNAs, sponging miRNAs to disrupt the balance between miRNA and target genes (164, 165), this regulation affects chondrocyte metabolism and intervenes in the OA process. For instance, Zhi et al. have found that in OA model tissues, the expression of the lncRNAs OIP5-AS1 and PGRN mRNA was down-regulated, and the expression of miR-29b-3p was upregulated. The expression of miR-29b-3p was negatively correlated with PGRN expression, OIP5-AS1 ameliorated chondrocyte apoptosis and inflammatory responses (166). After the knockdown of OIP5-AS1, the expression of miR-29b-3p was significantly increased in chondrocytes, and the inhibitory effect of si-OIP5-AS1 on cell proliferation was counteracted by the miR-29b-3p inhibitor (166). The expression of Bax was significantly reduced compared with that in the si-OIP5-AS1 group after co-transfection with miR-29b-3p inhibitor and si-OIP5-AS1, and the expression of inflammatory factors IL-6, IL-8, and TNF-α was significantly reduced compared with that in the si-OIP5-AS1 group (166). This suggests that OIP5-AS1 may regulate the proliferation, migration, apoptosis, and inflammatory response of chondrocytes via miR-29b-3p.

Furthermore, PGRN mRNA and protein expression decreased after the knockdown of OIP5-AS1, whereas PGRN mRNA expression increased after miR-29b-3p inhibition, knockdown of OIP5-AS1 ameliorated the inhibitory effect of miR-29b-3p on PGRN expression, suggesting that OIP5-AS1 can indirectly regulate PGRN by modulating miR-29b-3p, thus affecting chondrocyte proliferation, migration, apoptosis, and the inflammatory response (166). Therefore, OIP5-AS1 may indirectly regulate the expression of PGRN by sponging miR-29b-3p, promoting the viability and migration of chondrocytes in OA, inhibiting apoptosis and inflammation of chondrocytes in OA, and improving chondrocyte damage in OA. Therefore, the OIP5-AS1–miR-29b-3p–PGRN axis may serve as a novel target for OA therapy.

In summary, targeting non-coding RNAs to regulate PGRN expression represents a novel therapeutic approach for OA. However, recent studies have revealed that most non-coding RNAs lack tissue specificity, and their targeting may induce off-target side effects in other tissues (167, 168). Therefore, it is imperative not only to employ advanced screening technologies to identify tissue-specific non-coding RNAs, but also to utilise biological characteristics such as epigenetic synergism and innovative delivery systems. By establishing competing endogenous RNA networks and developing safe, precise, and efficient delivery platforms, we can mitigate the adverse effects associated with non-coding RNA therapies, thereby enhancing their therapeutic efficacy in disease treatment.

### 4.2 Mechanism of PGRN-derived protein Atsttrin in metabolic diseases

Atsttrin is an engineered protein derived from PGRN that consists of three PGRN fragments: half-unit GRNs A, C, and F and connexins P3, P4, and P5 (169). As the smallest molecule with PGRN function, Atsttrin does not exhibit oncogenic activity associated with endogenous PGRN, but it can significantly inhibit TNF-α-mediated inflammatory responses, demonstrating a potent anti-inflammatory effect (170). Additionally, Atsttrin can regulate bone homeostasis and cartilage repair. It has a longer half-life and a stronger therapeutic effect than PGRN, making it a promising bioactive factor with significant therapeutic effects in inflammatory bone diseases (171).

#### 4.2.1 Targeted therapeutic effect of Atsttrin on cartilage diseases

Atsttrin regulates cartilage metabolism by selectively binding to TNFR1/TNFR2. For instance, Wei et al. have found that Atsttrin could significantly reduce the expression of MMP-13, Col II, MCP-1, CCR-2, and IL-1β, effectively preventing COMP degradation and PGRN deficiency in OA progression (169). Further studies have shown that Atsttrin had little effect when TNFR1 was absent and was significantly reduced when TNFR2 was absent, indicating that this series of effects mediated by Atsttrin mainly depends on the TNFR2 pathway (169). It has also been found that Atsttrin strongly activated Akt signalling and slightly activated ERK1/2 signalling, whereas the activation of these signalling pathways was lost in TNFR2-deleted chondrocytes but maintained in TNFR1-deleted chondrocytes. This suggests that TNFR2 plays a major role in Atsttrin-mediated anabolic effects, additionally, when both ERK1/2 and Akt signalling inhibitors were used simultaneously, Atsttrin-mediated chondrocyte anabolic effects were almost eliminated, suggesting that Atsttrin promotes anabolism in chondrocytes by relying on the TNFR2-Akt-ERK1/2 signalling pathway (169).

The study has found that in TNFR1-deficient mice, Atsttrin treatment significantly upregulated the expression of cartilage marker genes such as Sox9, Col II, and aggrecan. However, in TNFR2-deficient mice, Atsttrin-mediated cartilage repair was significantly reduced (172). This suggests that Atsttrin can induce chondrocyte differentiation and stimulate chondrogenesis, however, this effect may depend on the TNFR2 pathway. The study has further found that Atsttrin treatment did not activate Erk1/2 signalling in WT, TNFR1-deficient, or TNFR2-deficient BMSCs. However, Atsttrin activated Akt signalling in WT and TNFR1-deficient BMSCs, but not in TNFR2-deficient chondrocytes, furthermore, the cartilage repair effect of Atsttrin was reversed by Akt inhibitors (172), indicating that cartilage repair and formation mediated by Atsttrin mainly depend on the TNFR2/Akt pathway.

Ding et al. have found that the expression of TNF-α in the discs and serum of patients with IDD was significantly increased. The increase in TNF-α expression significantly improved the expression of inflammatory factors, such as MMP-13, COX-2, iNOS, and IL-17, accelerated the loss of disc height and nucleus pulposus cells in the IVD, and strongly induced cartilage loss (173). This suggests that Atsttrin attenuates TNF-α-mediated catabolism, thereby reducing TNF-α-induced inflammation and preventing IDD. The study by Wang et al. also demonstrated that in biopsy samples from patients with degenerative nucleus pulposus and IDD mouse models, there was increased expression of TNF-α, TNFR1, TNFR2 and Bax, alongside reduced Bcl-2 expression (174). IDD mice with TNFR1 and TNFR2 deficiencies exhibited diminished disc area and enhanced endplate ossification, notably, TNFR2-deficient IDD mice showed more severe morphological disruption of intervertebral discs, with elevated MMP13, Cathepsin K and Bax expression, decreased Bcl-2 levels, and increased OC (174). Conversely, TNFR1-deficient mice displayed opposing trends. Atsttrin treatment significantly improved IDD scores in TNFR1-deficient mice, ameliorating disc space narrowing and nucleus pulposus signal loss, whereas no therapeutic effect was observed in TNFR2-deficient IDD mice (174). These findings indicate that TNFR1 and TNFR2 respectively mediate destructive and protective processes in IDD pathogenesis, with Atsttrin exerting its protective effects against IDD through TNFR2-dependent pathways.

In summary, our results showed that Atsttrin can accelerate cartilage repair and differentiation. These findings further confirm the positive protective effect of PGRN and its derived protein Atsttrin on cartilage degenerative diseases, such as OA, RA, and IVD degeneration.

#### 4.2.2 Targeted therapeutic effect of Atsttrin on OB differentiation inhibitory diseases

Atsttrin not only has a protective effect on cartilage but also has a therapeutic effect on OB differentiation inhibition in diseases. For instance, TNF-α treatment decreased the transcription levels of osteogenic marker genes such as ALP, COL1A1, OCN, and OPN under BMP-2 stimulation, whereas Atsttrin attenuated this inhibition mediated by TNF-α in a dose-dependent manner (175). Additionally, the study has also found that the alginate/hydroxyapatite deficient group showed a significant increase in the number of TNF-α positive cells and the expression of Runx2 (175). Therefore, it can be used as a new drug candidate for treating inflammatory bone diseases. Moradi et al. have found through *in vitro* studies that the Atsttrin-loaded hydrogel group significantly reduced the expression of IL-6 and iNOS in BMSCs and had higher expression levels of Glycosaminoglycans (GAGs) accumulation, calcium deposition, Col2a1, ACAN, Sox-9, Runx2, BMP-2, ALP, OCL, OCN, ALP, and Col-I than the hydrogel group (176). Further *in vivo* experiments found that in diabetic fracture mice, compared with the hydrogel group and PBS group, the hydrogel treatment loaded with Atsttrin reduced the callus and unmineralised cartilage at the fracture site and accelerated the healing speed of newly formed bone and bone space (176). This indicated that the presence of Atsttrin in the hydrogel formulation alleviated the TNF-α-induced inflammatory cascade, restored the balance of pro-inflammatory mediators, and stimulated cartilage repair and osteogenesis.

Taken together, the study of Atsttrin not only provides new insights into the role and mechanism of diabetic fracture healing but may also provide new and promising therapeutic intervention strategies for the development of therapeutic intervention strategies for various types of impaired fracture healing, especially diabetic fracture healing.

#### 4.2.3 Targeted therapy effect of Atsttrin on OB-OC coupling imbalance diseases

In addition to protecting the cartilage, Atsttrin has a positive preventive effect on OB-OC coupling. Atsttrin binds to both TNFR1 and TNFR2, inhibiting osteoclast differentiation and promoting osteoblast formation respectively (177). RANKL induces OC differentiation, while TNF-α significantly enhances RANKL-induced OC generated through upregulation of TRAP, CTSK and calcitonin receptor transcription. Atsttrin treatment markedly rescues TNF-α-potentiated OC formation, though it cannot directly inhibit RANKL-mediated OC differentiation (177). It was also found that TNFR1 RNAi inhibited TNFR1 expression, and knockdown of TNFR1, inhibited TNF-α-mediated OC generation, suggesting that Atsttrin inhibited TNF-α-induced OC generation through activation of TNFR1 (177).

The study further revealed that when BMSCs were cultured in OB medium with or without TNF-α, the presence of TNF-α significantly reduced the expression of ALP, Runx2, and Col-I. Notably, Atsttrin treatment effectively attenuated this inhibitory process. Moreover, knockdown of TNFR1 substantially reversed TNF-α-mediated OB suppression, while additional Atsttrin administration marginally enhanced osteoblastic activity, suggesting that Atsttrin likely inhibits TNF-α-mediated suppression of OB formation through activation of the TNFR1 pathway (177). Intriguingly, the investigation demonstrated that Atsttrin treatment alone did not alter the expression of osteogenic markers (Runx2, ALP, Col-1) under basal conditions, however, when combined with osteogenic culture medium, Atsttrin not only enhanced the transcriptional expression of osteogenic markers but also promoted both OB differentiation and functional activity, crucially, pharmacological inhibition of TNFR2 nearly abolished Atsttrin’s osteogenic effects, indicating that TNFR2 activation mediates Atsttrin-induced OB formation. Furthermore, mechanistic studies revealed that when OBs cultured in osteogenic medium (with or without Atsttrin) were subjected to PI3K/Akt pathway inhibition and/or ERK1/2 signalling blockade, the bone-forming effects mediated by Atsttrin were almost completely abrogated (177). These findings collectively demonstrate that Atsttrin facilitates OB formation through dual dependence on both TNFR2/Akt and TNFR2/ERK1/2 signalling cascades.

Taken together, these results show that although Atsttrin cannot directly induce OB formation, it can inhibit TNF-α-induced OC production by activating TNFR1, restore OB inhibition, and significantly promote OB formation by activating the TNFR2-Akt-ERK1/2 signalling pathway. This indicates that Atsttrin treatment may be a new strategy to regulate the OC/OB balance to maintain bone homeostasis.

In summary, the application of targeted PGRN and the PGRN-derived protein Atsttrin achieved a curative effect in the treatment of metabolic bone diseases (**Table 4**). However, its application in other metabolic diseases has not been reported. Therefore, researchers should not only increase the basic research scope of PGRN and its derivatives but also increase the development and application of PGRN in the technical field and combine theoretical research with practical applications. In recent years, the role of non-coding RNA in various diseases has become increasingly prominent. Therefore, it is necessary to strengthen research on non-coding RNA targeted regulation of PGRN in metabolic and other diseases. Additionally, the targeted regulatory effect of traditional Chinese medicine on PGRN has emerged, and traditional Chinese medicine has the characteristics of overall regulation and multi-point effects, which can take into account targeting and state-target binding along with modulating the state. Therefore, researchers should increase their research efforts on traditional Chinese medicine or traditional Chinese medicine in PGRN, develop more natural or plant-based inhibitors or agonists of PGRN, accelerate the pace of PGRN from theory to practice, and contribute to the development of medical and health undertakings and human health, making a modest contribution to health.

**Table 4:** Mechanism of targeted regulation of PGRN in metabolic diseases.

Target molecule	Molecular mechanism	Result	Disease	Refs
miR-29b-3p	Significantly inhibit the expression and release of PGRN, increase the expression of Bax, Caspase-3, MMP-1, MMP-13 and COL-X, reduce the transcription and secretion of col II, and inhibit the expression of Bcl-2. Recombinant PGRN can reduce the expression of Caspase-3 and Bax/Bcl-2 induced by miR-29b-3p.	Inhibition of chondrocyte apoptosis.	Cartilage and Bone homeostasis imbalance diseases Cartilage and Bone homeostasis imbalance diseases	(148)
miR-29b-3p	Matrine can reduce the production of IL-6, IL-1β and TNF-α in chondrocytes, inhibit the expression of miR-29b-3p and increase the expression of PGRN.	Improve the viability of chondrocytes, inhibit cell apoptosis, and reduce joint tissue damage.		(149)
LncRNA OIP5-AS1	Inhibits the expression of miR-29b-3p, down regulates the expression of Bax, IL-6, IL-8 and TNF-α, and up regulates the expression of PGRN by sponging miR-29b-3p.	Promote the proliferation and migration of chondrocytes, inhibit the apoptosis and inflammation of chondrocytes, and improve the injury of chondrocytes.		(154)
Atsttrin	By relying on TNFR2, Atsttrin strongly activated Akt pathway, slightly activated ERK1/2 pathway, and significantly increased the expression of cartilage marker genes Sox9, COL-II and Aggrecan.	Promote the anabolism of chondrocytes		(158, 159)
	Reduced the expression of inflammatory factors such as TNF-α, MMP-13, COX-2, iNOS and IL-17.	Alleviates the inflammatory reaction mediated by TNF-α and prevents intervertebral disc degeneration		(160)
	By interacting with TNFR2, the expression of TNF-α, TNFR1, TNFR2, Bax, MMP-13 and Cathepsin K was decreased, and the expression of Bcl-2 was increased	Intervertebral disc degeneration score was significantly improved, intervertebral disc space stenosis and NP signal and cartilage loss were weakened		(161)
	The transcription levels of ALP, COL1A1, OCN, OPN and Runx2 were up-regulated, and the formation and accumulation of TNF-α were decreased	Promote bone formation		(162)
	Significantly decreased the expression of TNF-α, IL-6 and iNOS in BMSCs, and increased the expression of GAGs accumulation, calcium deposition, Col2a1, ACAN, Sox-9, Runx2, BMP-2, ALP, OCL, OCN, ALP and Col-I	Alleviates the inflammatory cascade, restores the balance of pro-inflammatory mediators, and stimulates cartilage repair and bone formation		(163)
	By binding with TNFR1, Atsttrin down regulated the transcription levels of TNF-α, TRAP, CTSK and calcitonin receptor, and up-regulated the expressions of TNF-α, ALP, Runx2 and Col-I. By binding with TNFR2, Atsttrin activates Akt and ERK1/2 pathways and further increases the transcriptional expression of bone morphogenetic proteins Runx2, ALP and Col-I.	Inhibited OC differentiation, promoted ob differentiation, and maintained OB-OC balance		(164)

## 5 Summary and outlook

**Figure 11:**
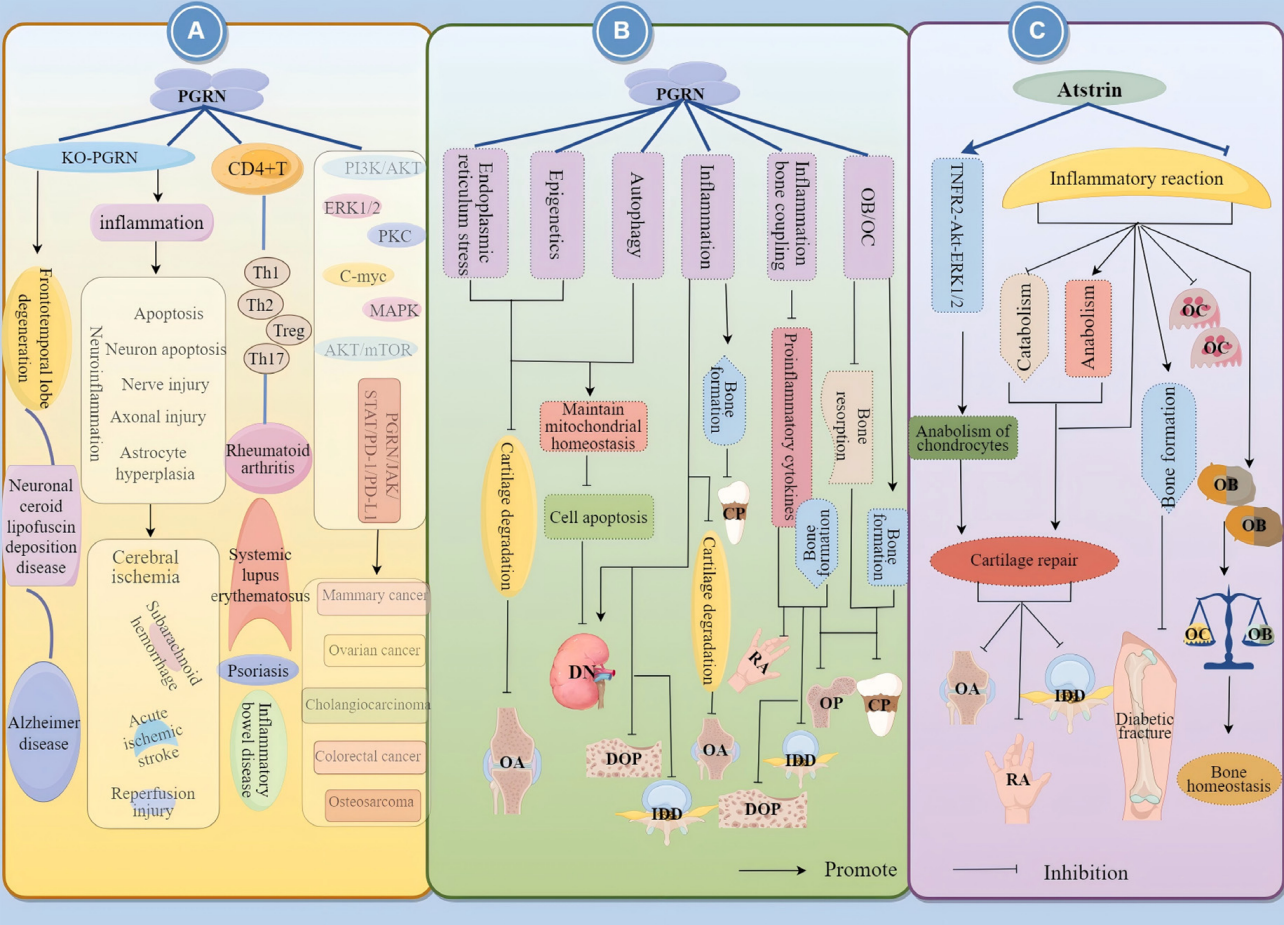
The role and mechanisms of action of PGRN (Progranulin) and its derivative Atsttrin in disease pathogenesis. **(A)** KO-PGRN not only directly contributes to neurodegenerative disorders such as frontotemporal lobar degeneration, neuronal ceroid lipofuscinosis, and Alzheimer’s disease, but also induces neuroinflammation, cellular apoptosis, axonal damage, astroglial hyperplasia, and neuronal death through inflammatory responses. This cascade mechanism predisposes individuals to cerebral ischaemia/reperfusion injury, subarachnoid haemorrhage, and acute ischaemic stroke. Furthermore, PGRN plays a significant role in immune-inflammatory pathologies including rheumatoid arthritis, inflammatory bowel disease, psoriasis, and systemic lupus erythematosus by modulating the differentiation of CD4+ T cells into Th1, Th2, Th17, and Treg subsets. Additionally, PGRN mediates multiple signalling pathways - including PI3K/AKT, ERK1/2, PKC, C-myc, MAPK, AKT/mTOR, and PGRN/JAK/STAT/PD-1/PD-L1 - thereby influencing the progression of various malignancies such as colorectal carcinoma, osteosarcoma, ovarian cancer, cholangiocarcinoma, and breast cancer. **(B)** Furthermore, PGRN exerts protective effects against OA by suppressing cartilage degradation through endoplasmic reticulum stress, epigenetic regulation, and autophagy. PGRN also maintains mitochondrial homeostasis and inhibits cellular apoptosis via autophagy, thereby demonstrating protective functions in DN. Through inflammatory responses, PGRN exhibits dual regulatory roles: while directly promoting DN progression and suppressing IDD and DOP, it concurrently protects against OA and CP by inhibiting cartilage degradation and enhancing bone formation. Additionally, PGRN modulates the inflammation-bone coupling axis to suppress inflammatory reactions and promote osteogenesis, consequently inhibiting the development of RA, DOP, OP, and IDD. By regulating OB-OC equilibrium, PGRN inhibits bone resorption while stimulating bone formation, thereby conferring protection against CP and OP. **(C)** Finally, the PGRN derivative Atsttrin demonstrates therapeutic potential through dual mechanisms: it promotes anabolic processes and cartilage repair in OA, RA, and IDD by activating the TNFR2-Akt-ERK1/2 pathway while suppressing inflammatory responses and catabolic activity. Concurrently, Atsttrin enhances bone formation, inhibits OC differentiation, and modulates OB-OC balance, offering protective effects against bone destructive disorders and pathologies involving bone homeostasis dysregulation.

This article reviews the molecular mechanisms of PGRN in metabolic diseases such as OA, RA, IVD degeneration, OP, EP, and diabetes-related complications (**Figure 11**). First, the structure and function of PGRN and the relationship between PGRN and various diseases are discussed. Second, the changes in the expression and molecular mechanisms of PGRN in these metabolic diseases are examined. Finally, the application of the targeted regulation of PGRN and PGRN-derived proteins in metabolic diseases is introduced.

Recent studies have confirmed that PGRN, a pleiotropic cell growth factor, has tissue- and system- specificity (178, 179). It plays a role in inflammation, cartilage repair, and the maintenance of bone homeostasis in inflammatory diseases, OA, and bone homeostasis imbalance (10, 107, 108). However, in cancerous diseases, high expression of PGRN promotes the differentiation, proliferation, and angiogenesis of tumour cells, accelerating the invasion of cancer cells (63). Further studies have shown that the pleiotropic property of PGRN may selectively compete with PGRN for binding to TNFRs (TNFR1 and TNFR2), inhibit the biological function of TNF-α, and subsequently directly or indirectly activate/inhibit the inflammatory response and other effects triggered by inflammation (31). Conversely, it may be related to the intervention of PGRN in macrophage differentiation, thereby regulating the relevant immune system (180). Therefore, the inflammatory response caused by TNF-α remains the core of disease development. These functions of PGRN provide valuable insights into its critical role in cellular life and suggest that PGRN is a potential target for the regulation of human health and diseases.

Given the importance of PGRN in various cellular and biological processes, its impact on various diseases has also received attention. As mentioned above, PGRN is involved in regulating the occurrence and development of diseases, including neurodegenerative diseases such as Parkinson’s disease, Creutzfeldt-Jakob disease, motor neuron disease, and Alzheimer’s disease (55–58), and immune-inflammatory diseases such as RA, inflammatory bowel disease, psoriasis, and SLE (60, 65–68). The role of PGRN as a pro-inflammatory regulator of inflammation requires special attention.

Additionally, the interventional effects of PGRN on some disease processes are related to the regulation of specific signalling pathways and molecular phenotypes. For instance, studies have shown that PGRN intervenes in OA by regulating molecular phenotypes such as autophagy, ER stress, and acetylation (10, 51, 77–80, 84, 87, 88, 94, 95). However, recent studies have found that mitochondrial homeostasis imbalance (mitochondrial autophagy and biogenesis) (181), macrophage polarisation (182–184), ferroptosis (185), copper death (186), pyroptosis (187), and the recently discovered ammonia death (188) are closely related to disease occurrence, development, and prognosis. Therefore, is PGRN related to phenotypes and signalling pathways such as mitochondrial homeostasis imbalance, macrophage polarisation, ferroptosis, copper death, pyroptosis, and ammonia death? PGRN can affect the progression and prognosis of metabolic diseases such as OA, RA, IVD degeneration, OP, EP, and diabetes-related complications by regulating or mediating these phenotypes and signalling pathways.

Recently, the incidence of chronic metabolic diseases has increased. Although conventional Western medicine has achieved certain curative effects, it also has a series of side effects, including long treatment cycles, high costs, and low patient acceptance and recognition, To overcome this problem, it is imperative to explore safe, effective, and low-cost therapeutic drugs as well as other treatment methods. With the development of network pharmacology, molecular biology, and bioinformatics technology, it has been found that traditional Chinese medicine has the characteristics of overall regulation, multi-point effects, safety, and low cost. Patients have a high degree of recognition and acceptance of traditional Chinese medicine treatment or conditioning, and traditional Chinese medicine treatment has achieved gratifying results for several diseases (189, 190). Therefore natural/plant activators or inhibitors of PGRN should be developed to accelerate the progress of PGRN research, from basic research to clinical applications. However, it is worth noting that, based on the tissue and system specificity of PGRN, when developing natural/plant activators or inhibitors of PGRN, attention must be focused on the tissue type and system of the target disease.

As evidenced above, PGRN exhibits tissue-specific characteristics and interacts through this modality with pathways including endoplasmic reticulum stress, autophagy, inflammatory responses, and epigenetic regulation, thereby establishing an intricate mechanistic network that contributes to the maintenance of metabolic homeostasis. Consequently, modulation of PGRN expression profiles in distinct tissues; comprehensive elucidation of its pathophysiological roles and molecular regulatory mechanisms in metabolic diseases; and development of tissue-targeted PGRN derivatives with enhanced specificity, may be a novel therapeutic strategy for the treatment of metabolic diseases.
